# Supplementary material for: Sensory processing in humans and mice fluctuates between external and internal modes
Source: PLoS Biol. 2023 Dec 8;21(12):e3002410. doi: 10.1371/journal.pbio.3002410 (PMC10732408; doi:10.1371/journal.pbio.3002410)
Supplement: S1 Text — In this supplemental file, we show that internal mode processing is driven by choice history as opposed to stimulus history, that fluctuations between internal and external mode modulate perceptual performance beyond the effect of general response biases, that internal mode is characterized by lower thresholds as well as by history-dependent changes in biases and lapses, and that internal mode processing can not be reduced to insufficient task familiarity. (PDF) [file pbio.3002410.s001.pdf]

S1 Text: Sensory processing in humans and mice fluctuates between external and internal modes

Authors:

Veith Weilnhammer[1,2,3], Heiner Stuke[1,2], Kai Standvoss[1], Philipp Sterzer[4]

Affiliations:

[1] Department of Psychiatry, Charité-Universitätsmedizin Berlin, corporate member of Freie Universität Berlin and Humboldt-Universität zu Berlin, 10117 Berlin, Germany

[2] Berlin Institute of Health, Charité-Universitätsmedizin Berlin and Max Delbrück Center, 10178 Berlin, Germany

[3] Helen Wills Neuroscience Institute, University of California Berkeley, USA

[4] Department of Psychiatry (UPK), University of Basel, Switzerland

Corresponding Author:

Veith Weilnhammer, Helen Wills Neuroscience Institute, University of California Berkeley, USA, email: veith.weilnhammer@gmail.com

1 Supplemental Text

1.1 Internal mode processing is driven by choice history as opposed to stimulus history

The main manuscript reports the effects of perceptual history, which we defined as the impact of the choice at the preceding trial on the choice at the current trial (henceforth *choice history*). *Stimulus history*, which is defined as the impact of the stimulus presented at the preceding trial on the choice at the present trial, represents an alternative approach to this. Here, we compare the effects of choice history to the effects of stimulus history.

We observed a significant bias toward stimulus history (humans: 49.76% ± 0.1% of trials, $\beta = 1.26 \pm 0.94$, T(373.62) = 1.34, p = 0.18; mice: 51.11% ± 0.08% of trials, T(164) = 13.4, p $= 3.86 \times 10^{-28}$). The bias toward stimulus history was smaller than the bias toward choice history (humans: $\beta = -3.53 \pm 0.5$, T(66.53) = −7.01, p $= 1.48 \times 10^{-9}$; mice: T(164) = -17.21, p $= 1.43 \times 10^{-38}$).

The attraction of choices toward both preceding choices and stimuli is expected, as perception was *stimulus-congruent* on approximately 75% of trials, causing choices and stimuli to be highly correlated. We therefore compared the effects of choice history and stimulus history after *stimulus-incongruent* (i.e., *error*) trials, since those trials lead to opposite predictions regarding the perceptual choice at the subsequent trial.

As expected from the findings presented in the main manuscript, perceptual choices were attracted toward perceptual choices when the inducing trial was stimulus-incongruent (i.e., a positive effect of choice history; humans: $\beta = 0.19 \pm 1.4 \times 10^{-4}$, z $= 1.36 \times 10^{3}$, p $< 2.2 \times 10^{-308}$: mice: $\beta = 0.92 \pm 0.01$, z = 88.82, p $< 2.2 \times 10^{-308}$). By contrast, perceptual choices tended to be repelled away from the stimulus presented at preceding stimulus-incongruent trial (i.e., a negative effect of stimulus history; humans: $\beta = -0.19 \pm 0.01$, z = −16.47, p $= 5.99 \times 10^{-61}$: mice: $\beta = -0.92 \pm 0.01$, z = −88.76, p $< 2.2 \times 10^{-308}$). This repulsion of

choices away from stimuli presented at stimulus-incongruent trials confirmed that choices (which are anti-correlated to stimuli at stimulus-incongruent trials) were the primary driver of attracting serial effects in perception.

In sum, the above results suggest that, in both humans and mice, serial dependencies were better explained by the effects of choice history as opposed to the effects of stimulus history. This aligns with a result recently published for the IBL database, where mice were shown to follow an *action-kernel* as opposed to a *stimulus-kernel* model when integrating information across trials[81].

1.2 Fluctuations between internal and external mode modulate perceptual performance beyond the effect of general response biases

The hypothesis that perception cycles through opposing internally- and externally-biased modes is motivated by the assumption that recurring intervals of stronger perceptual history temporally reduce the participants' sensitivity to external information. Importantly, the history-dependent biases that characterize internal mode processing must be differentiated from general response biases. In binary perceptual decision-making, general response biases are defined by a propensity to choose one of the two outcomes more often than the alternative. Indeed, human participants selected the more frequent of the two possible outcomes in 58.71% ± 0.22% of trials, and mice selected the more frequent of the two possible outcomes in 54.6% ± 0.3% of trials.

Two caveats have to be considered to make sure that the effect of history-congruence is distinct from the effect of general response biases. First, history-congruent states become more likely for larger response biases that cause an increasing imbalance in the likelihood of the two outcomes (humans: $\beta = 0.24 \pm 6.93 \times 10^{-4}$, $T(2.09 \times 10^6) = 342.43$, $p < 2.2 \times 10^{-308}$; mice: $\beta = 0.15 \pm 8.25 \times 10^{-4}$, $T(1.32 \times 10^6) = 181.93$, $p < 2.2 \times 10^{-308}$). One may thus

ask whether the autocorrelation of history-congruence could be entirely driven by general response biases.

Importantly, our autocorrelation analyses account for general response biases by computing group-level autocorrelations (Figs 2-4B) relative to randomly permuted data (i.e., by subtracting the autocorrelation of randomly permuted data from the raw autocorrelation curve). This precludes that general response biases contribute to the observed autocorrelation of history-congruence (see S5 Fig for a visualization of the correction procedure for simulated data with general response biases ranging from 60 to 90%).

Second, it may be argued that fluctuations in perceptual performance may be solely driven by ongoing changes in the strength of general response biases. To assess the links between dynamic fluctuations in stimulus-congruence on the one hand and history-congruence as well as general response bias on the other hand, we computed all variables as dynamic probabilities in sliding windows of ± 5 trials (Fig 1C). Linear mixed effects modeling indicated that fluctuations in history-congruent biases were larger in amplitude than the corresponding fluctuations in general response biases in humans ($\beta_0 = 0.03 \pm 7.34 \times 10^{-3}$, $T(64.94) = 4.46$, $p = 3.28 \times 10^{-5}$), but slightly smaller in mice ($\beta_0 = -5.26 \times 10^{-3} \pm 4.67 \times 10^{-4}$, $T(2.12 \times 10^3) = -11.28$, $p = 1.02 \times 10^{-28}$).

Crucially, ongoing fluctuations in history-congruence had a significant negative effect on stimulus-congruence (humans: $\beta_1 = -0.05 \pm 5.63 \times 10^{-4}$, $T(2.1 \times 10^6) = -84.21$, $p < 2.2 \times 10^{-308}$; mice: $\beta_1 = -0.12 \pm 7.17 \times 10^{-4}$, $T(1.34 \times 10^6) = -168.39$, $p < 2.2 \times 10^{-308}$) beyond the effect of ongoing changes in general response biases (humans: $\beta_2 = -0.06 \pm 5.82 \times 10^{-4}$, $T(2.1 \times 10^6) = -103.51$, $p < 2.2 \times 10^{-308}$; mice: $\beta_2 = -0.03 \pm 6.94 \times 10^{-4}$, $T(1.34 \times 10^6) = -48.14$, $p < 2.2 \times 10^{-308}$). In sum, the above control analyses confirmed that, in both humans and mice, the observed influence of preceding choices on perceptual decision-making cannot be reduced to general response biases.

1.3 Internal mode is characterized by lower thresholds as well as by history-dependent changes in biases and lapses

Random or stereotypical responses may provide an alternative explanation for the reduced sensitivity to external sensory information that we attribute to internal mode processing. To test this hypothesis, we asked whether history-independent changes in biases and lapses may provide an alternative explanation of the reduced sensitivity during internal mode.

To this end, we estimated full and history-conditioned psychometric curves to investigate how internal and external mode relate to biases (i.e., the horizontal position of the psychometric curve), lapses (i.e., the asymptotes of the psychometric curve) and thresholds (i.e., 1/sensitivity, estimated from the slope of the psychometric curve). We used a maximum likelihood procedure to predict trial-wise choices y ($y = 0$ and $y = 1$ for outcomes A and B respectively) from the choice probabilities y_p. y_p was computed from the difficulty-weighted inputs s_w via a parametric error function defined by the parameters γ (lower lapse), δ (upper lapse), μ (bias) and t (threshold; see Methods for details):

$$y_p = \gamma + (1 - \gamma - \delta) * (erf(\frac{s_w + \mu}{t}) + 1)/2 \quad (1)$$

Under our main hypothesis that periodic reductions in sensitivity to external information are driven by increases in the impact of perceptual history, one would expect (i) a history-dependent increase in biases and lapses (effects of perceptual history), and (ii), a history-independent increase in threshold (reduced sensitivity to external information). Conversely, if what we identified as internal mode processing was in fact driven by random choices, one would expect (i), a history-independent increase in lapses (choice randomness), (ii), no change in bias (no effect of perceptual history), and (iii), reduced thresholds (reduced sensitivity to external information).

1.3.1 Humans

Across all data provided by the Confidence database[20] (i.e., irrespective of the preceding perceptual choice y_{t-1}), biases μ were distributed around zero (-0.05 ± 0.03; $\beta_0 = 7.37 \times 10^{-3}$ ± 0.09, T(36.8) = 0.08, p = 0.94; S6A-B Fig, upper panel). When conditioned on perceptual history, biases μ varied according to the preceding perceptual choice, with negative biases for $y_{t-1} = 0$ (-0.22 ± 0.04; $\beta_0 = 0.56 \pm 0.12$, T(43.39) = 4.6, p = 3.64×10^{-5}; S6A-B Fig, upper panel) and positive biases for $y_{t-1} = 1$ (0.29 ± 0.03; $\beta_0 = 0.56 \pm 0.12$, T(43.39) = 4.6, p = 3.64×10^{-5}; S6A-B Fig, lower panel). Absolute biases $|\mu|$ were larger in internal mode (1.84 ± 0.03) as compared to external mode (0.86 ± 0.02; $\beta_0 = -0.62 \pm 0.07$, T(45.62) = −8.38, p = 8.59×10^{-11}; controlling for differences in lapses and thresholds).

Lower and upper lapses amounted to $\gamma = 0.13 \pm 2.83 \times 10^{-3}$ and $\delta = 0.1 \pm 2.45 \times 10^{-3}$ (S6A Fig, S6C and S6D). Lapses were larger in internal mode ($\gamma = 0.17 \pm 3.52 \times 10^{-3}$, $\delta = 0.14 \pm 3.18 \times 10^{-3}$) as compared to external mode ($\gamma = 0.1 \pm 2.2 \times 10^{-3}$, $\delta = 0.08 \pm 2 \times 10^{-3}$; $\beta_0 = -0.05 \pm 5.73 \times 10^{-3}$, T(47.03) = −9.11, p = 5.94×10^{-12}; controlling for differences in biases and thresholds).

Conditioning on the previous perceptual choice revealed that the between-mode difference in lapse was not general, but depended on perceptual history: For $y_{t-1} = 0$, only higher lapses δ differed between internal and external mode ($\beta_0 = -0.1 \pm 9.58 \times 10^{-3}$, T(36.87) = −10.16, p = 3.06×10^{-12}), whereas lower lapses γ did not ($\beta_0 = 0.01 \pm 7.77 \times 10^{-3}$, T(33.1) = 1.61, p = 0.12). Vice versa, for $y_{t-1} = 1$, lower lapses γ differed between internal and external mode ($\beta_0 = -0.11 \pm 0.01$, T(40.11) = −9.59, p = 6.14×10^{-12}), whereas higher lapses δ did not ($\beta_0 = 0.01 \pm 7.74 \times 10^{-3}$, T(33.66) = 1.58, p = 0.12).

Thresholds t were estimated at 3 ± 0.06 (S6A Fig and S6E). Thresholds t were larger in internal mode (3.66 ± 0.09) as compared to external mode (2.02 ± 0.03; $\beta_0 = -1.77 \pm 0.25$, T(50.45) = −7.14, p = 3.48×10^{-9}; controlling for differences in biases and lapses). In contrast to the bias μ and the lapse rates γ and δ, thresholds t were not modulated by

perceptual history ($\beta_0 = 0.04 \pm 0.06$, T(2.97×10^3) = 0.73, p = 0.47).

1.3.2 Mice

When estimated based on the full dataset provided in the IBL database[21] (i.e., irrespective of the preceding perceptual choice y_{t-1}), biases μ were distributed around zero ($3.87 \times 10^{-3} \pm 9.81 \times 10^{-3}$; T(164) = 0.39, p = 0.69; S7A-B Fig, upper panel). When conditioned on the preceding perceptual choice, biases were negative for $y_{t-1} = 0$ ($-0.02 \pm 8.7 \times 10^{-3}$; T(164) = -1.99, p = 0.05; S7A-B Fig, middle panel) and positive for $y_{t-1} = 1$ ($0.02 \pm 9.63 \times 10^{-3}$; T(164) = 1.91, p = 0.06; S7A-B Fig, lower panel). As in humans, mice showed larger biases during internal mode ($0.14 \pm 7.96 \times 10^{-3}$) as compared to external mode ($0.07 \pm 8.7 \times 10^{-3}$; $\beta_0 = -0.18 \pm 0.03$, T = -6.38, p = 1.77×10^{-9}; controlling for differences in lapses and thresholds).

Lower and upper lapses amounted to $\gamma = 0.1 \pm 4.35 \times 10^{-3}$ and $\delta = 0.11 \pm 4.65 \times 10^{-3}$ (S7A Fig, S7C, and S7D). Lapse rates were higher in internal mode ($\gamma = 0.15 \pm 5.14 \times 10^{-3}$, $\delta = 0.16 \pm 5.79 \times 10^{-3}$) as compared to external mode ($\gamma = 0.06 \pm 3.11 \times 10^{-3}$, $\delta = 0.07 \pm 3.34 \times 10^{-3}$; $\beta_0 = -0.11 \pm 4.39 \times 10^{-3}$, T = -24.8, p = 4.91×10^{-57}; controlling for differences in biases and thresholds).

For $y_{t-1} = 0$, the difference between internal and external mode was more pronounced for higher lapses δ (T(164) = 21.44, p = 1.93×10^{-49}). Conversely, for $y_{t-1} = 1$, the difference between internal and external mode was more pronounced for lower lapses γ (T(164) = -18.24, p = 2.68×10^{-41}). In contrast to the human data, higher lapses δ and lower lapses γ were significantly elevated during internal mode irrespective of the preceding perceptual choice (higher lapses δ for $y_{t-1} = 1$: T(164) = -2.65, p = 8.91×10^{-3}; higher lapses δ for $y_{t-1} = 0$: T(164) = -28.29, p = 5.62×10^{-65}; lower lapses γ for $y_{t-1} = 1$: T(164) = -32.44, p = 2.92×10^{-73}; lower lapses γ for $y_{t-1} = 0$: T(164) = -2.5, p = 0.01).

In mice, thresholds t amounted to $0.15 \pm 6.52 \times 10^{-3}$ (S7A Fig and S7E) and were higher

in internal mode (0.27 ± 0.01) as compared to external mode ($0.09 \pm 4.44 \times 10^{-3}$; $\beta_0 = -0.28 \pm 0.04$, T = -7.26, p = 1.53×10^{-11}; controlling for differences in biases and lapses). Thresholds t were not modulated by perceptual history (T(164) = 0.94, p = 0.35).

In sum, the above analyses showed that, in both humans and mice, internal and external mode differ with respect to biases, lapses and thresholds. Internally-biased processing was characterized by higher thresholds, indicating a reduced sensitivity to sensory information, as well as by larger biases and lapses. Importantly, between-mode differences in biases and lapses strongly depended on perceptual history. This confirmed that internal mode processing cannot be explained solely on the ground of a general (i.e., history-independent) increase in lapses or bias indicative of random of stereotypical responses.

1.4 Internal mode processing can not be reduced to insufficient task familiarity

It may be assumed that participants tend to repeat preceding choices when they are not yet familiar with the experimental task, leading to history-congruent choices that are caused by insufficient training. To assess this alternative explanation, we contrasted the correlates of bimodal inference with training effects in humans and mice.

1.4.1 Humans

In the Confidence database[20], training effects were visible from RTs that were shortened by increasing exposure to the task ($\beta = -7.53 \times 10^{-5} \pm 6.32 \times 10^{-7}$, T($1.81 \times 10^{6}$) = -119.15, p $< 2.2 \times 10^{-308}$). Intriguingly, however, history-congruent choices became more frequent with increased exposure to the task ($\beta = 3.6 \times 10^{-5} \pm 2.54 \times 10^{-6}$, z = 14.19, p = 10^{-45}), speaking against the proposition that insufficient training induces seriality in response behavior.

1.4.2 Mice

As in humans, it is an important caveat to consider whether the observed serial dependencies in mice reflect a phenomenon of perceptual inference, or, alternatively, an unspecific strategy that occurs at the level of reporting behavior. We reasoned that, if mice indeed tended to repeat previous choices as a general response pattern, history effects should decrease during training of the perceptual task. We therefore analyzed how stimulus- and history-congruent perceptual choices evolved across sessions in mice that, by the end of training, achieved proficiency (i.e., stimulus-congruence $\geq 80\%$) in the *basic* task of the IBL dataset[21].

Across sessions, we found that stimulus-congruent perceptual choices became more frequent ($\beta = 0.34 \pm 7.13 \times 10^{-3}$, $T(8.51 \times 10^{3}) = 47.66$, $p < 2.2 \times 10^{-308}$) and TDs were progressively shortened ($\beta = -22.14 \pm 17.06$, $T(1.14 \times 10^{3}) = -1.3$, $p < 2.2 \times 10^{-308}$). Crucially, the frequency of history-congruent perceptual choices also increased during training ($\beta = 0.13 \pm 4.67 \times 10^{-3}$, $T(8.4 \times 10^{3}) = 27.04$, $p = 1.96 \times 10^{-154}$; S8 Fig).

Within individual session, longer task exposure was associated with an increase in history-congruence ($\beta = 3.6 \times 10^{-5} \pm 2.54 \times 10^{-6}$, $z = 14.19$, $p = 10^{-45}$) and a decrease in TDs ($\beta = -0.1 \pm 3.96 \times 10^{-3}$, $T(1.34 \times 10^{6}) = -24.99$, $p = 9.45 \times 10^{-138}$). In sum, these findings strongly argue against the proposition that mice show biases toward perceptual history due to an unspecific response strategy.
