# Supplementary figures and images for: Sensory processing in humans and mice fluctuates between external and internal modes
Source: PLoS Biol. 2023 Dec 8;21(12):e3002410. doi: 10.1371/journal.pbio.3002410 (PMC10732408; doi:10.1371/journal.pbio.3002410)

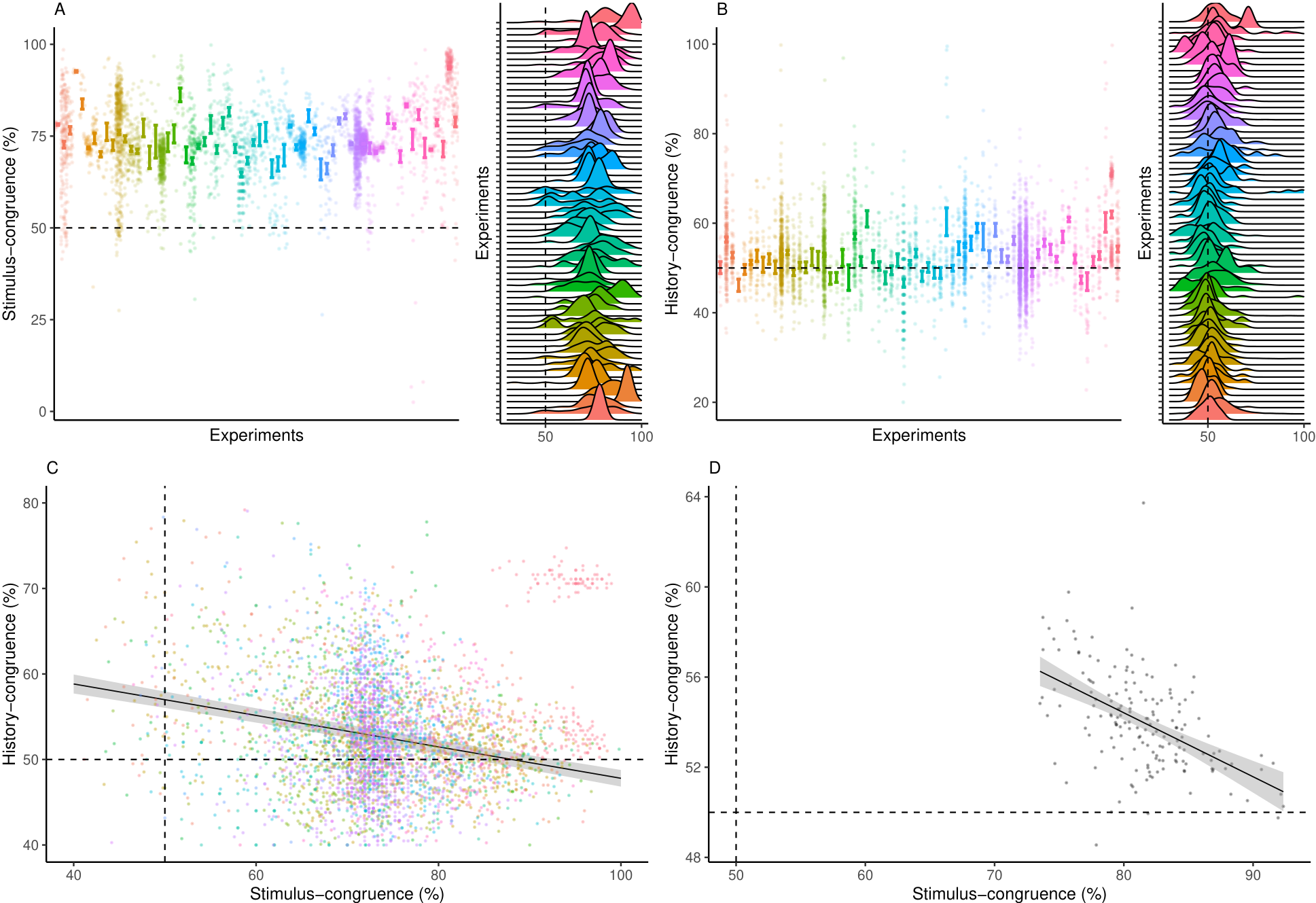

Supplement: S1 Fig — (A) Stimulus-congruent choices in humans amounted to 73.46% ± 0.15% of trials and were highly consistent across the experiments selected from the Confidence database. (B) History-congruent choices in humans amounted to 52.7% ± 0.12% of trials. In analogy to stimulus-congruence, the prevalence of history-congruence was highly consistent across the experiments selected from the Confidence database. A percentage of 48.48% of experiments showed significant (p < 0.05) biases toward preceding choices, whereas 2 of the 66 of the included experiments showed significant repelling biases. (C) In humans, we found an enhanced impact of perceptual history in participants who were less sensitive to external sensory information (T(4.3×10^3^) = −14.27, p = 3.78×10^−45^), suggesting that perception results from the competition of external with internal information. (D) In analogy to humans, mice that were less sensitive to external sensory information showed stronger biases toward perceptual history (T(163) = −7.52, p = 3.44×10^−12^, Pearson correlation). (TIFF) [file pbio.3002410.s002.tiff]

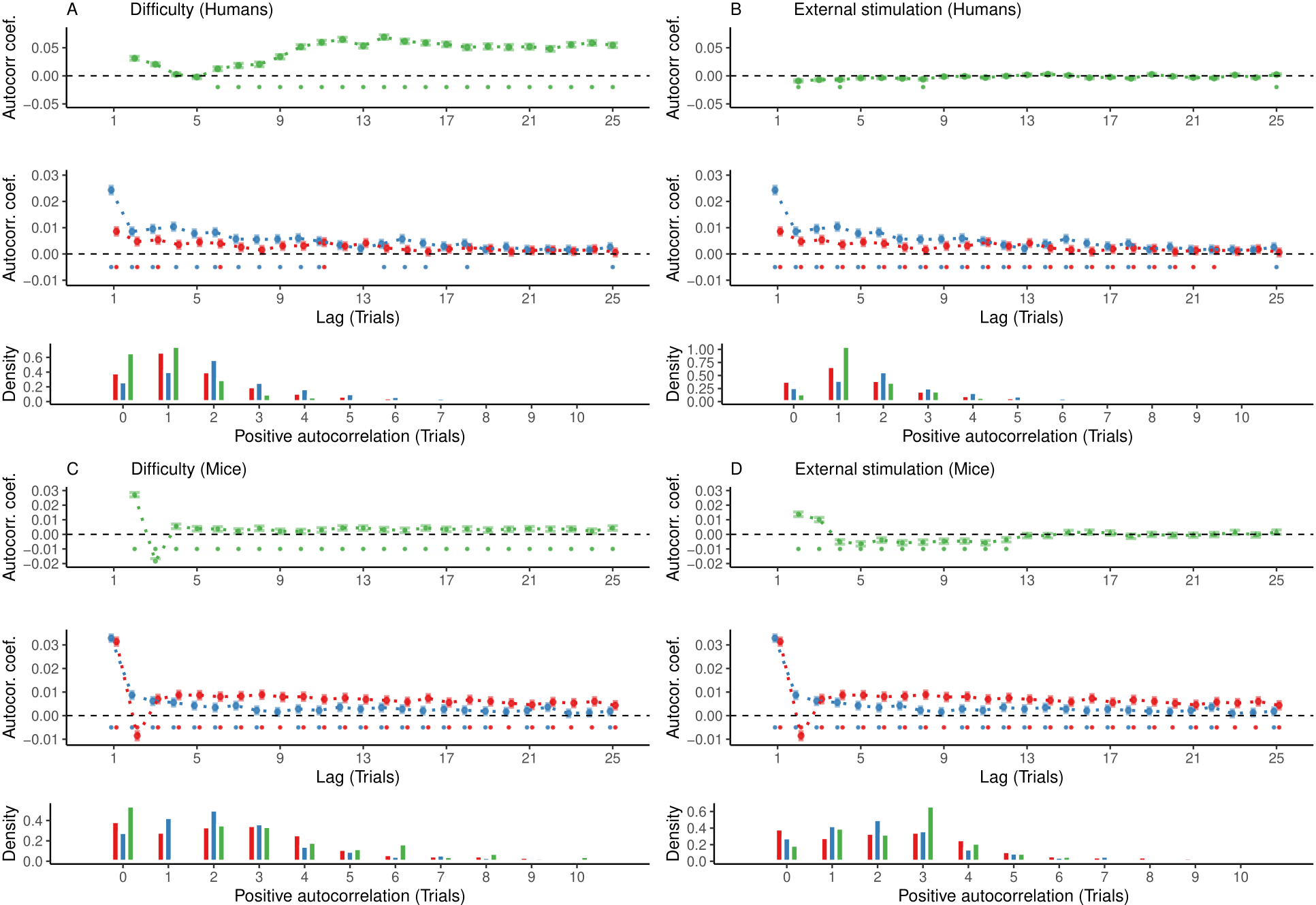

Supplement: S2 Fig — In this study, we found highly significant autocorrelations of stimulus- and history-congruence in humans as well as in mice, while controlling for task difficulty and the sequence of external stimulation. Here, we confirm that the autocorrelations of stimulus- and history-congruence were not a trivial consequence of the experimental design or the addition of task difficulty and external stimulation as control variables in the computation of group-level autocorrelations. (A) In humans, task difficulty (in green) showed a significant autocorrelation starting at the fifth trial (upper panel, dots at the bottom indicate intercepts ≠ 0 in trial-wise linear mixed effects modeling at p < 0.05). When controlling for task difficulty only, linear mixed effects modeling indicated a significant autocorrelation of stimulus-congruence (in red) for the first 3 consecutive trials (middle panel). Around 20% of trials within the displayed time window remained significantly autocorrelated. The autocorrelation of history-congruence (in blue) remained significant for the first 11 consecutive trials (64% significantly autocorrelated trials within the displayed time window). At the level of individual participants, the autocorrelation of task difficulty exceeded the respective autocorrelation of randomly permuted within a lag of 21.66 ± 8.37×10−3 trials (lower panel). (B) In humans, the sequence of external stimulation (i.e., which of the 2 binary outcomes was supported by the presented stimuli; depicted in green) was negatively autocorrelated for 1 trial. When controlling for the autocorrelation of external stimulation only, stimulus-congruence remained significantly autocorrelated for 22 consecutive trials (88% of trials within the displayed time window; lower panel) and history-congruence remained significantly autocorrelated for 20 consecutive trials (84% of trials within the displayed time window). At the level of individual participants, the autocorrelation of external stimulation [file pbio.3002410.s003.tiff]

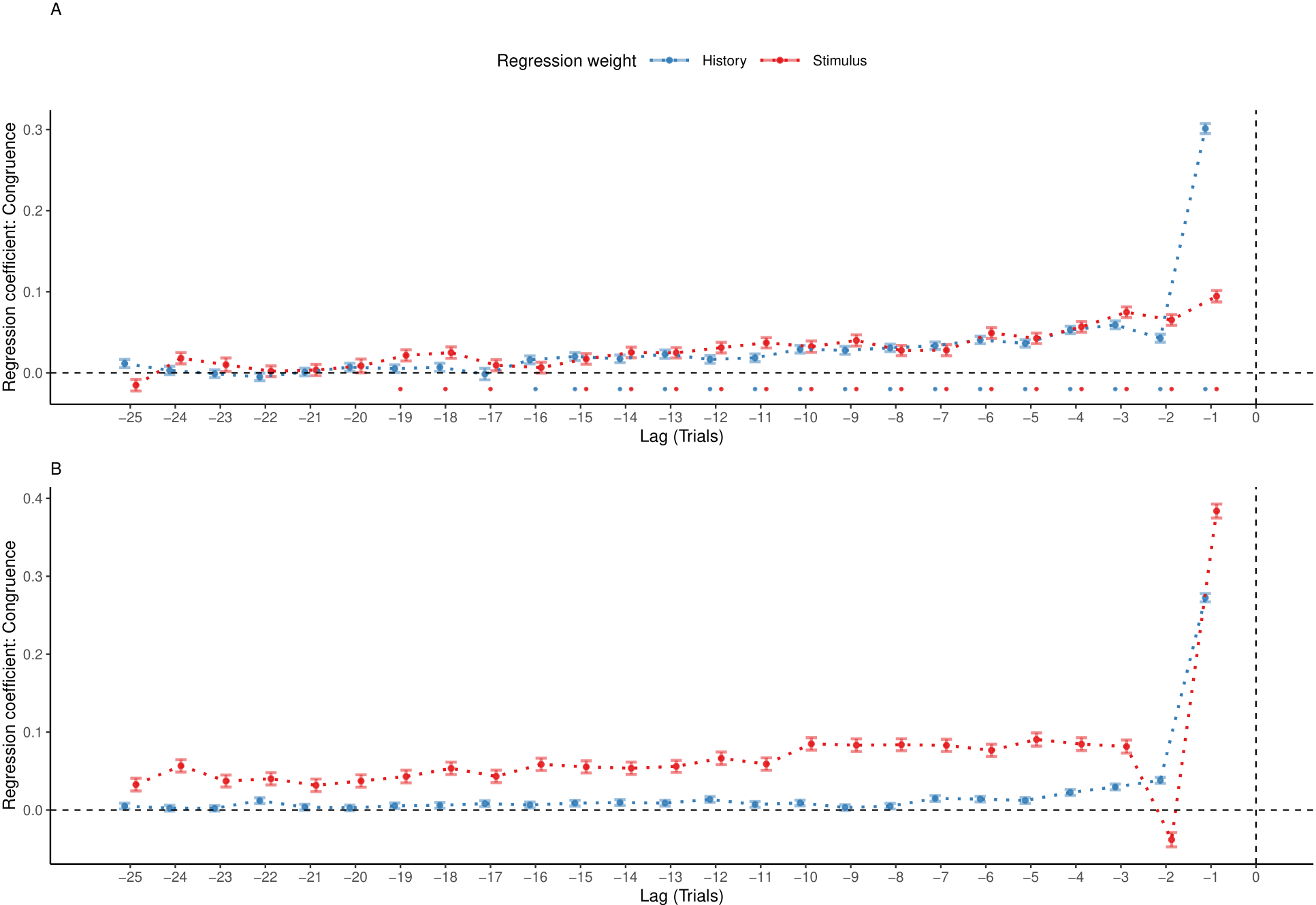

Supplement: S3 Fig — (A) As an alternative to group-level autocorrelation coefficients, we used trial-wise logistic regression to quantify serial dependencies in stimulus- and history-congruence. This analysis predicted stimulus- and history-congruence at the index trial (trial t = 0, vertical line) based on stimulus- and history-congruence at the 100 preceding trials. Mirroring the shape of the group-level autocorrelations, trial-wise regression coefficients (depicted as mean ± SEM, dots mark trials with regression weights significantly greater than 0 at p < 0.05) increased toward the index trial t = 0 for the human data. (B) Following our results in human data, regression coefficients that predicted history-congruence at the index trial (trial t = 0, vertical line) increased exponentially for trials closer to the index trial in mice. In contrast to history-congruence, stimulus-congruence showed a negative regression weight (or autocorrelation coefficient; Fig 3B) at trial −2. This was due to the experimental design (see also the autocorrelations of difficulty and external stimulation in S2 Fig): When mice made errors at easy trials (contrast ≥ 50%), the upcoming stimulus was shown at the same spatial location and at high contrast. This increased the probability of stimulus-congruent perceptual choices after stimulus-incongruent perceptual choices at easy trials, thereby creating a negative regression weight (or autocorrelation coefficient) of stimulus-congruence at trial −2. (TIFF) [file pbio.3002410.s004.tiff]

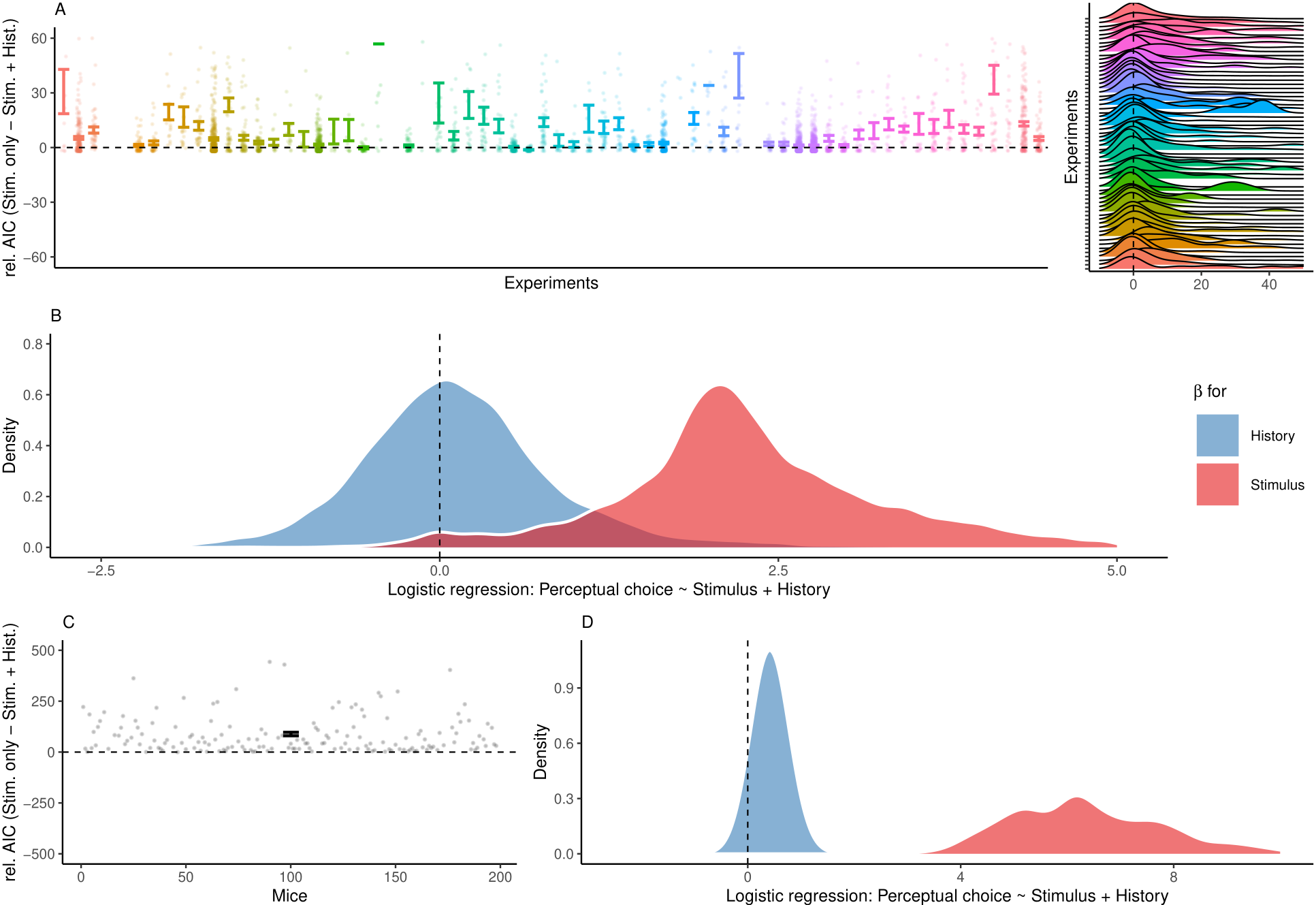

Supplement: S4 Fig — (A) To ensure that perceptual history played a significant role in perception despite the ongoing stream of external information, we tested whether human perceptual decision-making was better explained by the combination of external and internal information or, alternatively, by external information alone. To this end, we compared AIC between logistic regression models that predicted trial-wise perceptual responses either by both current external sensory information and the preceding percept or by external sensory information alone (values above 0 indicate a superiority of the full model). With high consistency across the experiments selected from the Confidence Database, this model comparison confirmed that perceptual history contributed significantly to perception (difference in AIC = 8.07 ± 0.53, T(57.22) = 4.1, p = 1.31×10^−4^). (B) Participant-wise regression coefficients amount to 0.18 ± 0.02 for the effect of perceptual history and 2.51 ± 0.03 for external sensory stimulation. (C) In mice, an AIC-based model comparison indicated that perception was better explained by logistic regression models that predicted trial-wise perceptual responses based on both current external sensory information and the preceding percept (difference in AIC = 88.62 ± 8.57, T(164) = −10.34, p = 1.29×10^−19^). (D) In mice, individual regression coefficients amounted to 0.42 ± 0.02 for the effect of perceptual history and 6.91 ± 0.21 for external sensory stimulation. (TIFF) [file pbio.3002410.s005.tiff]

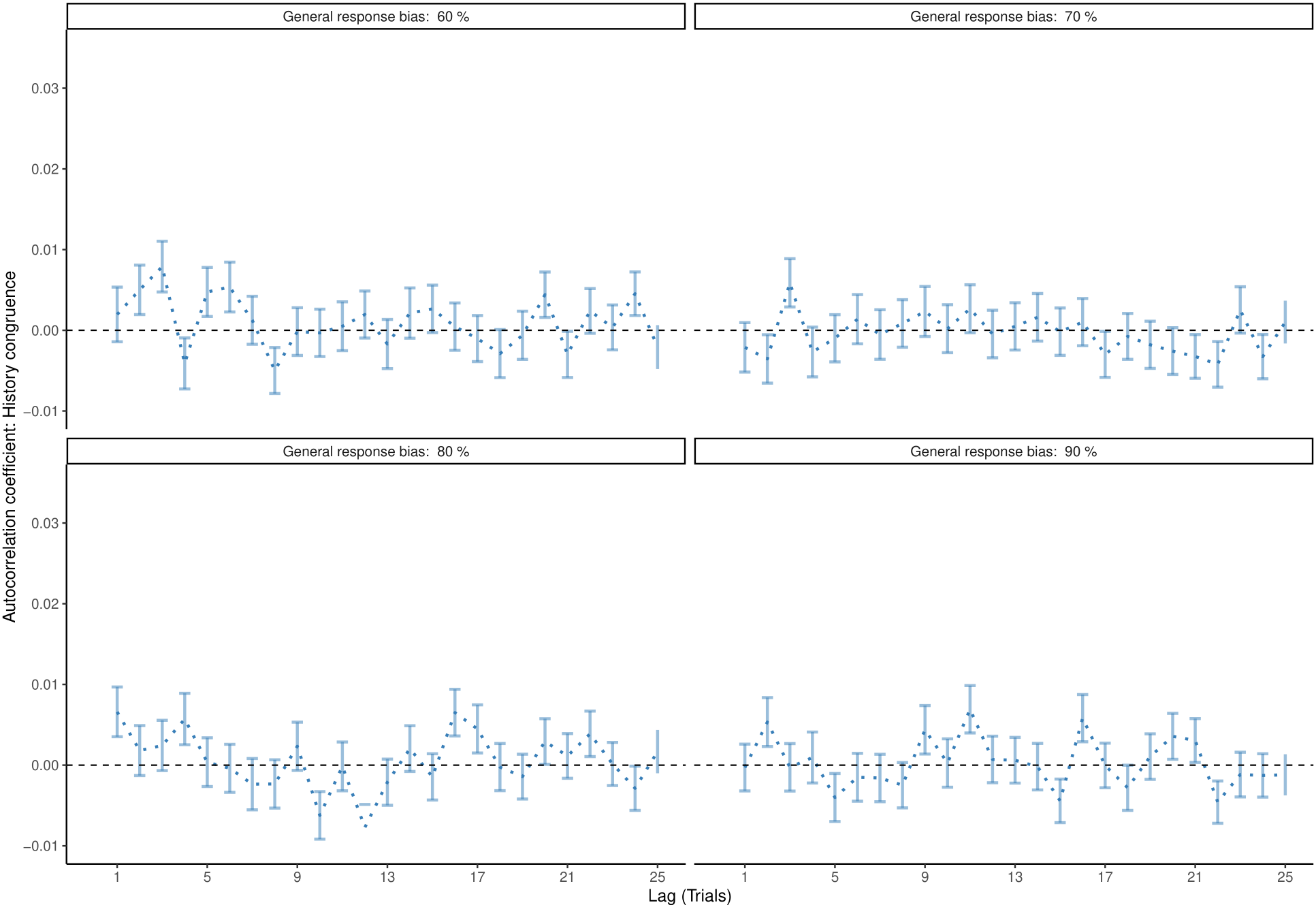

Supplement: S5 Fig — Here, we ask whether the autocorrelation of history-congruence (as shown in Figs 2–3C) may be driven by general response biases (i.e., a general propensity to choose one of the 2 possible outcomes more frequently than the alternative). To this end, we generated sequences of 100 perceptual choices with general response biases ranging from 60% to 90% for 1,000 simulated participants each. We then computed the autocorrelation of history-congruence for these simulated data. Crucially, we used the correction procedure that is applied to the autocorrelation curves shown in this manuscript: All reported autocorrelation coefficients are computed relative to the average autocorrelation coefficients obtained for 100 iterations of randomly permuted trial sequences. The above simulation show that this correction procedure removes any potential contribution of general response biases to the autocorrelation of history-congruence. This indicates that the autocorrelation of history-congruence (as shown in Figs 2–3C) is not driven by general response biases that were present in the empirical data at a level of 58.71% ± 0.22% in humans and 54.6% ± 0.3% in mice. (TIFF) [file pbio.3002410.s006.tiff]

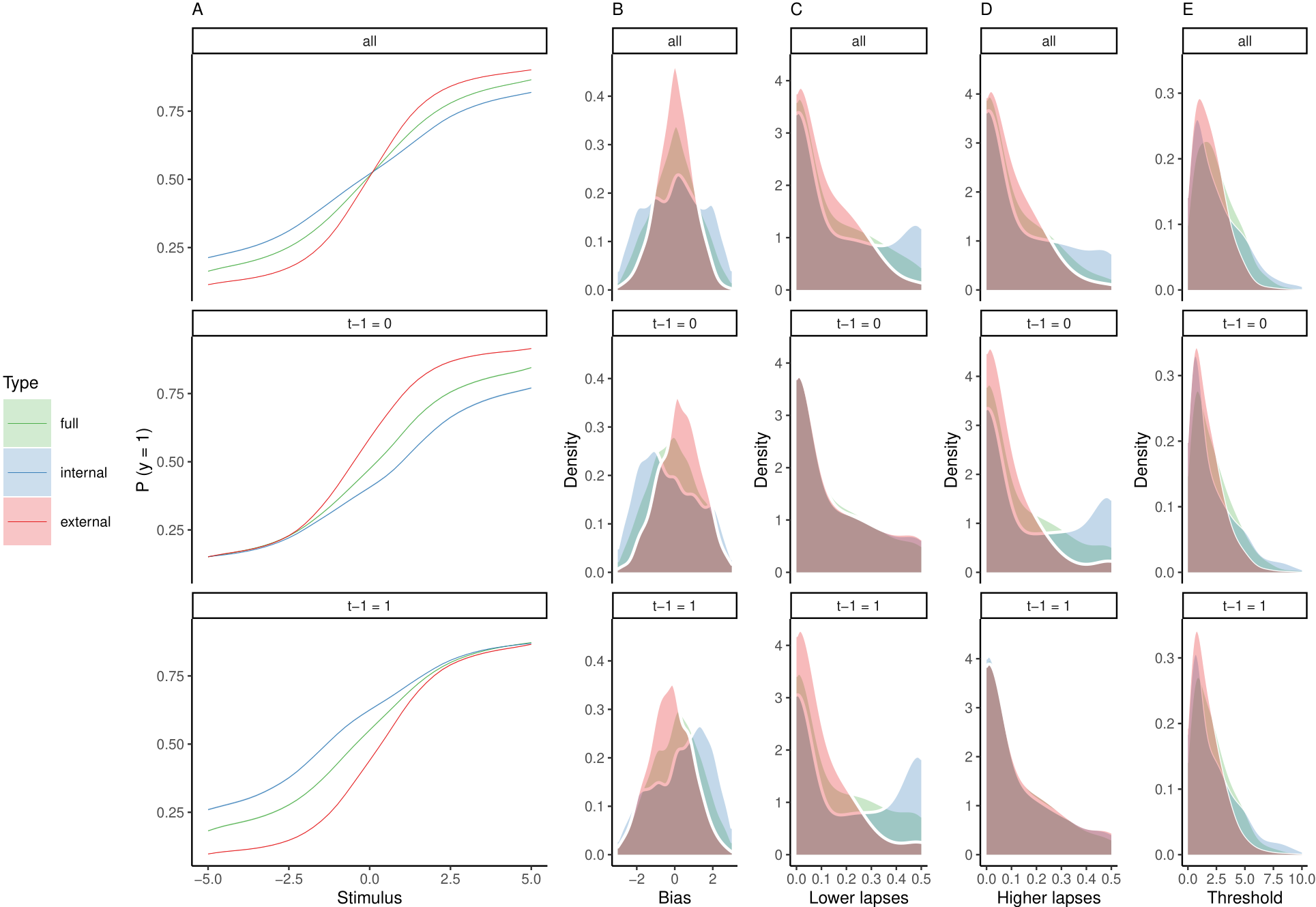

Supplement: S6 Fig — (A) Here, we show average psychometric functions for the full dataset (upper panel) and conditioned on perceptual history (yt−1 = 1 and yt−1 = 0; middle and lower panel) across modes (green line) and for internal mode (blue line) and external mode (red line) separately. (B) Across the full dataset, biases μ were distributed around 0 (β0 = 7.37×10^−3^ ± 0.09, T(36.8) = 0.08, p = 0.94; upper panel), with larger absolute biases |μ| for internal as compared to external mode (β0 = −0.62 ± 0.07, T(45.62) = −8.38, p = 8.59×10^−11^; controlling for differences in lapses and thresholds). When conditioned on perceptual history, we observed negative biases for yt−1 = 0 (β0 = 0.56 ± 0.12, T(43.39) = 4.6, p = 3.64×10^−5^; middle panel) and positive biases for yt−1 = 1 (β0 = 0.56 ± 0.12, T(43.39) = 4.6, p = 3.64×10^−5^; lower panel). (C) Lapse rates were higher in internal mode as compared to external mode (β0 = −0.05 ± 5.73×10^−3^, T(47.03) = −9.11, p = 5.94×10^−12^; controlling for differences in biases and thresholds; see upper panel and subplot D). Importantly, the between-mode difference in lapses depended on perceptual history: We found no significant difference in lower lapses γ for yt−1 = 0 (β0 = 0.01 ± 7.77×10^−3^, T(33.1) = 1.61, p = 0.12; middle panel), but a significant difference for yt−1 = 1 (β0 = −0.11 ± 0.01, T(40.11) = −9.59, p = 6.14×10^−12^; lower panel). (D) Conversely, higher lapses δ were significantly increased for yt−1 = 0 (β0 = −0.1 ± 9.58×10^−3^, T(36.87) = −10.16, p = 3.06×10^−12^; middle panel), but not for yt−1 = 1 (β0 = 0.01 ± 7.74×10^−3^, T(33.66) = 1.58, p = 0.12; lower panel). (E) The thresholds t were larger in internal as compared to external mode (β0 = −1.77 ± 0.25, T(50.45) = −7.14, p = 3.48×10^−9^; controlling for differences in biases and lapses) and were not modulated by perceptual history (β0 = 0.04 ± 0.06, T(2.97×10^3^) = 0.73, p = 0.47). (TIFF) [file pbio.3002410.s007.tiff]

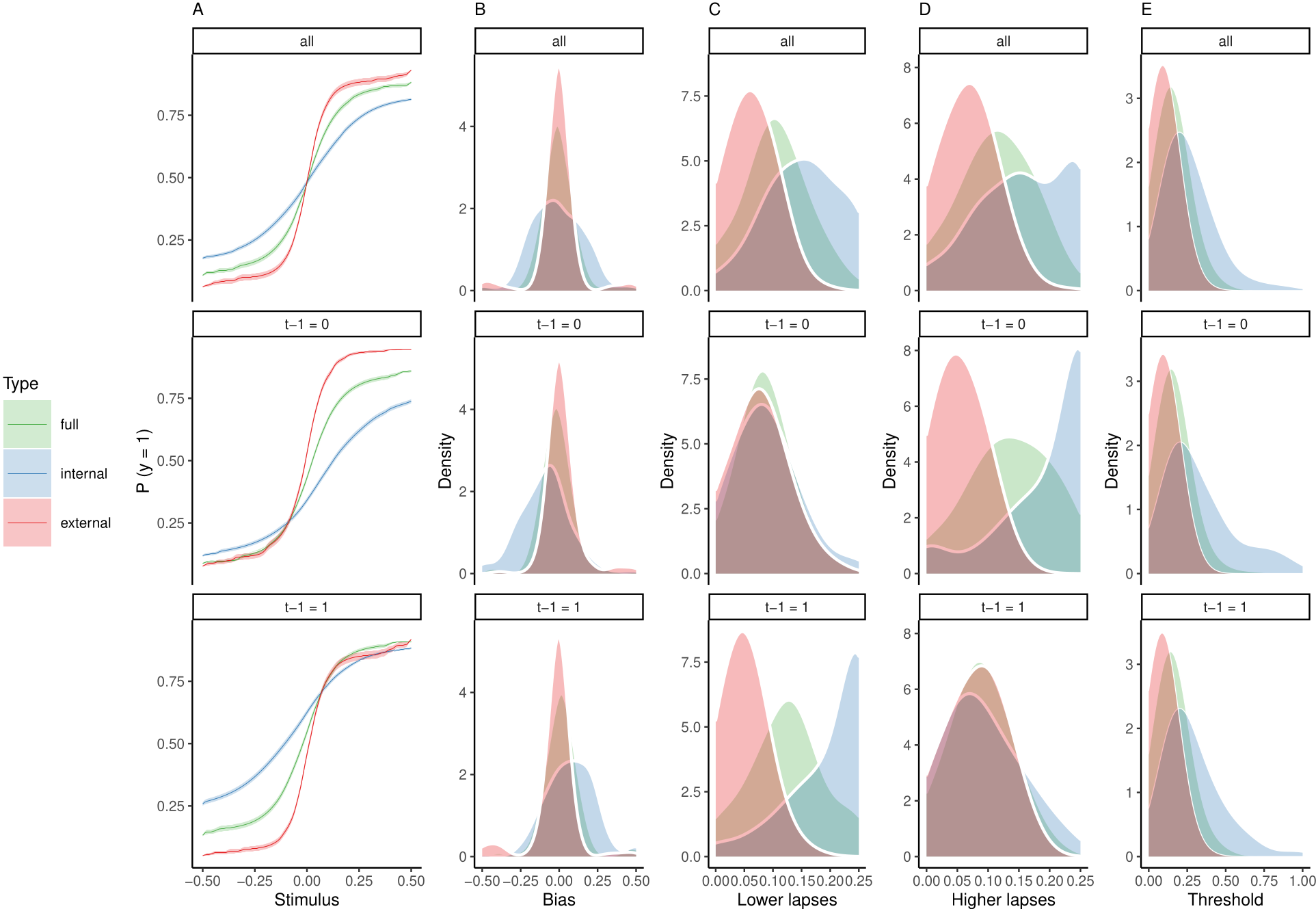

Supplement: S7 Fig — (A) Here, we show average psychometric functions for the full IBL dataset (upper panel) and conditioned on perceptual history (yt−1 = 1 and yt−1 = 0; middle and lower panel) across modes (green line) and for internal mode (blue line) and external mode (red line) separately. (B) Across the full dataset, biases μ were distributed around 0 (T(164) = 0.39, p = 0.69; upper panel), with larger absolute biases |μ| for internal as compared to external mode (β0 = −0.18 ± 0.03, T = −6.38, p = 1.77×10^−9^; controlling for differences in lapses and thresholds). When conditioned on perceptual history, we observed negative biases for yt−1 = 0 (T(164) = -1.99, p = 0.05; middle panel) and positive biases for yt−1 = 1 (T(164) = 1.91, p = 0.06; lower panel). (C) Lapse rates were higher in internal as compared to external mode (β0 = −0.11 ± 4.39×10^−3^, T = −2.48, p = 4.91×10^−57^; controlling for differences in biases and thresholds; upper panel, see subplot D). For yt−1 = 1, the difference between internal and external mode was more pronounced for lower lapses γ (T(164) = −18.24, p = 2.68×10^−41^) as compared to higher lapses δ (see subplot D). In mice, lower lapses γ were significantly elevated during internal mode irrespective of the preceding perceptual choice (middle panel: lower lapses γ for yt−1 = 0; T(164) = −2.5, p = 0.01, lower panel: lower lapses γ for yt−1 = 1; T(164) = −32.44, p = 2.92×10^−73^). (D) For yt−1 = 0, the difference between internal and external mode was more pronounced for higher lapses δ (T(164) = 21.44, p = 1.93×10^−49^; see subplot C). Higher lapses were significantly elevated during internal mode irrespective of the preceding perceptual choice (middle panel: higher lapses δ for yt−1 = 0; T(164) = −28.29, p = 5.62×10^−65^ lower panel: higher lapses δ for yt−1 = 1; T(164) = −2.65, p = 8.91×10^−3^). (E) Thresholds t were higher in internal as compared to external mode (β0 = −0.28 ± 0.04, T = −7.26, p = 1.53×10^−11^; controlling for differences in biases and lapses) and were [file pbio.3002410.s008.tiff]

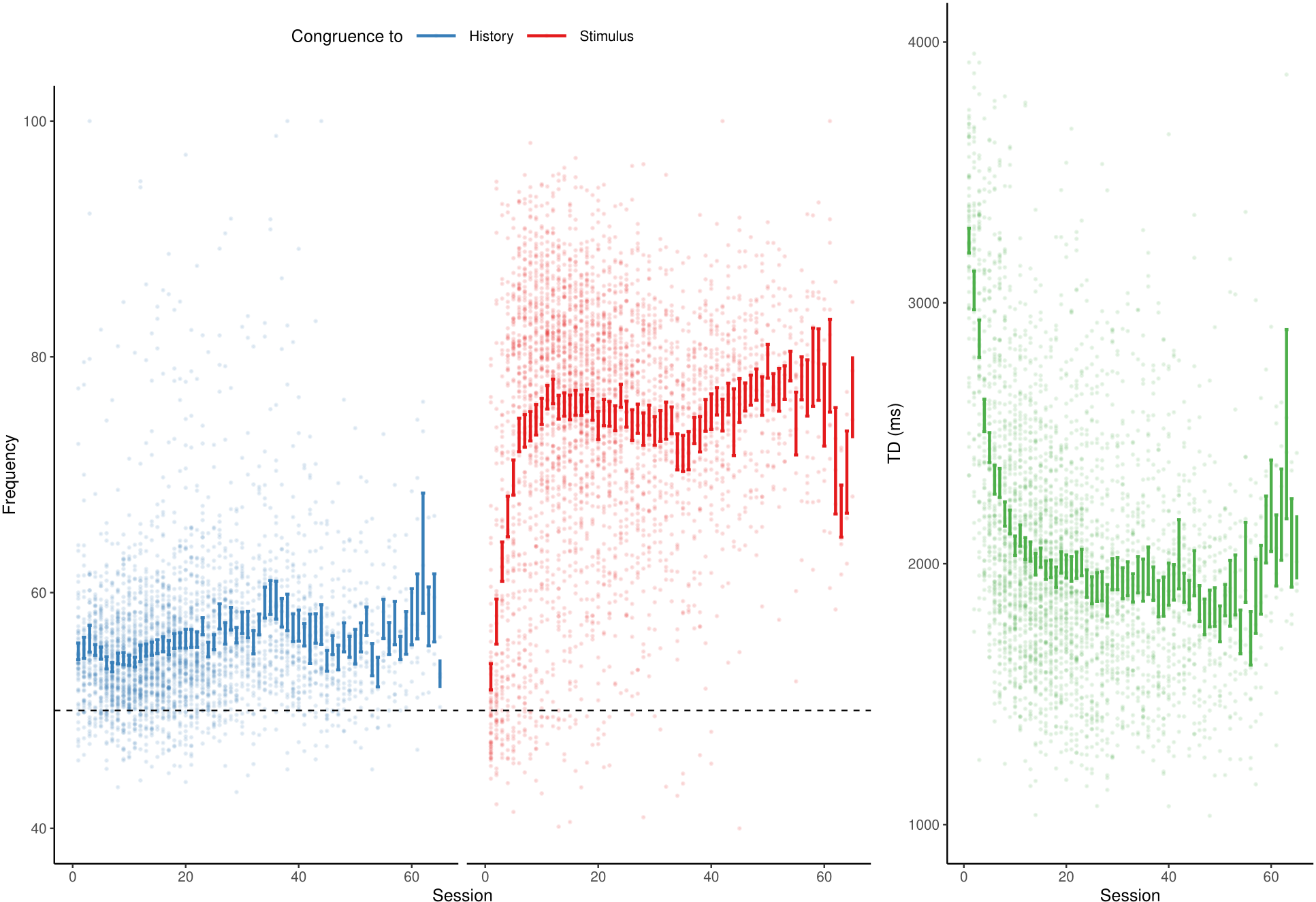

Supplement: S8 Fig — Here, we depict the progression of history- and stimulus-congruence (depicted in blue and red, respectively; left panel) as well as TDs (in green; right panel) across training sessions in mice that achieved proficiency (i.e., stimulus-congruence ≥ 80%) in the basic task of the IBL dataset. We found that both history-congruent perceptual choices (β = 0.13 ± 4.67×10^−3^, T(8.4×10^3^) = 27.04, p = 1.96×10−154) and stimulus-congruent perceptual choices (β = 0.34 ± 7.13×10^−3^, T(8.51×10^3^) = 47.66, p < 2.2×10−308) became more frequent with training. As in humans, mice showed shorter TDs with increased exposure to the task (β = −22.14 ± 17.06, T(1.14×10^3^) = −1.3, p < 2.2×10−308). (TIFF) [file pbio.3002410.s009.tiff]

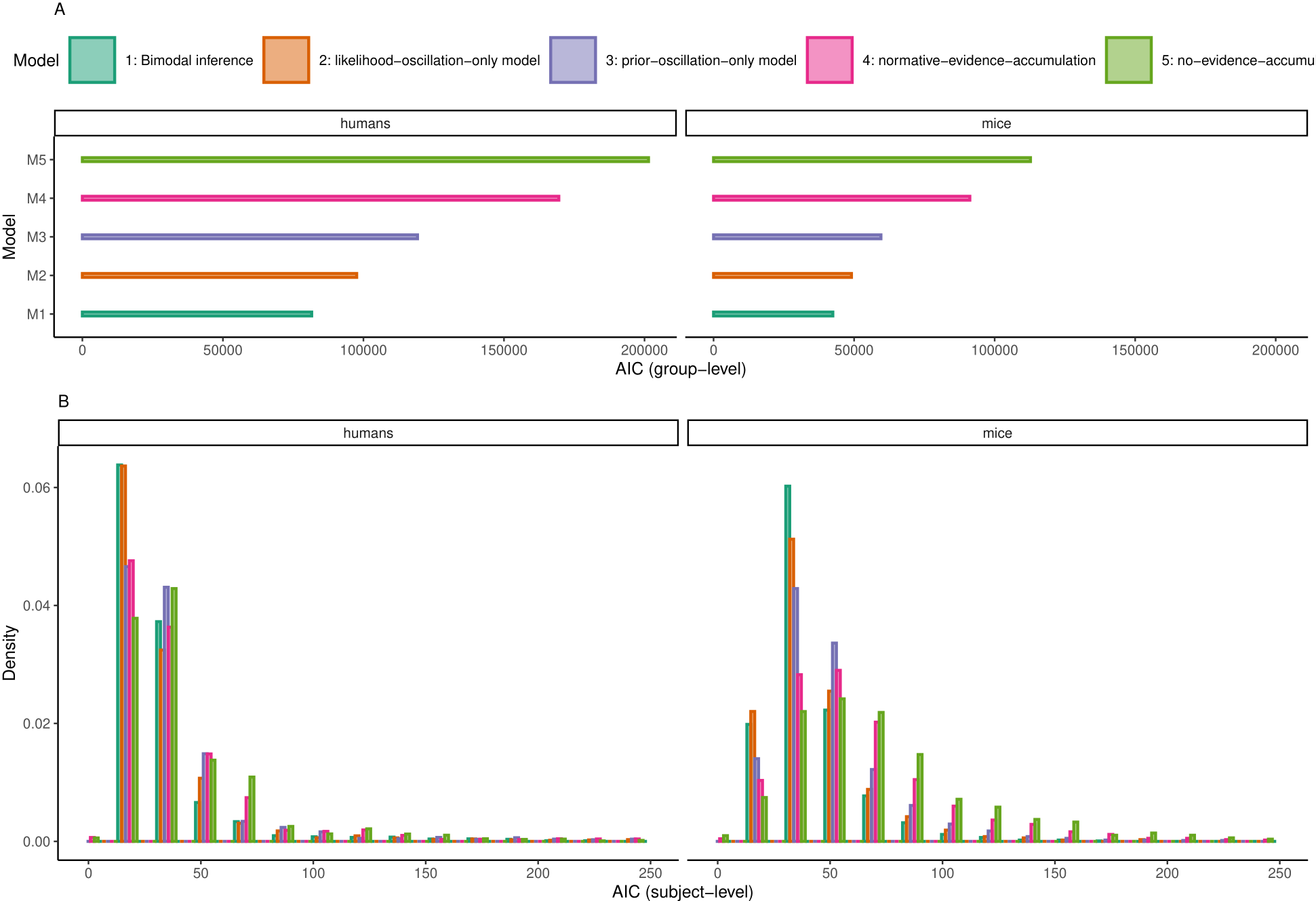

Supplement: S9 Fig — (A) Group-level AIC. The bimodal inference model (M1) achieved the lowest AIC across the full model space (AIC1 = 8.16×10^4^ in humans and 4.24×10^4^ in mice). Model M2 (AIC2 = 9.76×10^4^ in humans and 4.91×10^4^ in mice) and Model M3 (AIC3 = 1.19×10^5^ in humans and 5.95×10^4^ in mice) incorporated only oscillations of either likelihood or prior precision. Model M4 (AIC4 = 1.69×10^5^ in humans and 9.12×10^4^ in mice) lacked any oscillations of likelihood and prior precision and corresponded to the normative model proposed by Glaze and colleagues [51]. In model M5 (AIC4 = 2.01×10^5^ in humans and 1.13×10^5^ in mice), we furthermore removed the integration of information across trials, such that perception depended only in incoming sensory information. (B) Subject-level AIC. Here, we show the distribution of AIC values at the subject level. AIC for the bimodal inference model tended to be smaller than AIC for the comparator models (statistical comparison to the second-best model M2 in humans: β = −1.71 ± 0.19, T(8.57×10^3^) = −8.85, p = 1.06×10^−18^; mice: T(1.57×10^3^) = -3.08, p = 2.12×10^−3^). (TIFF) [file pbio.3002410.s010.tiff]

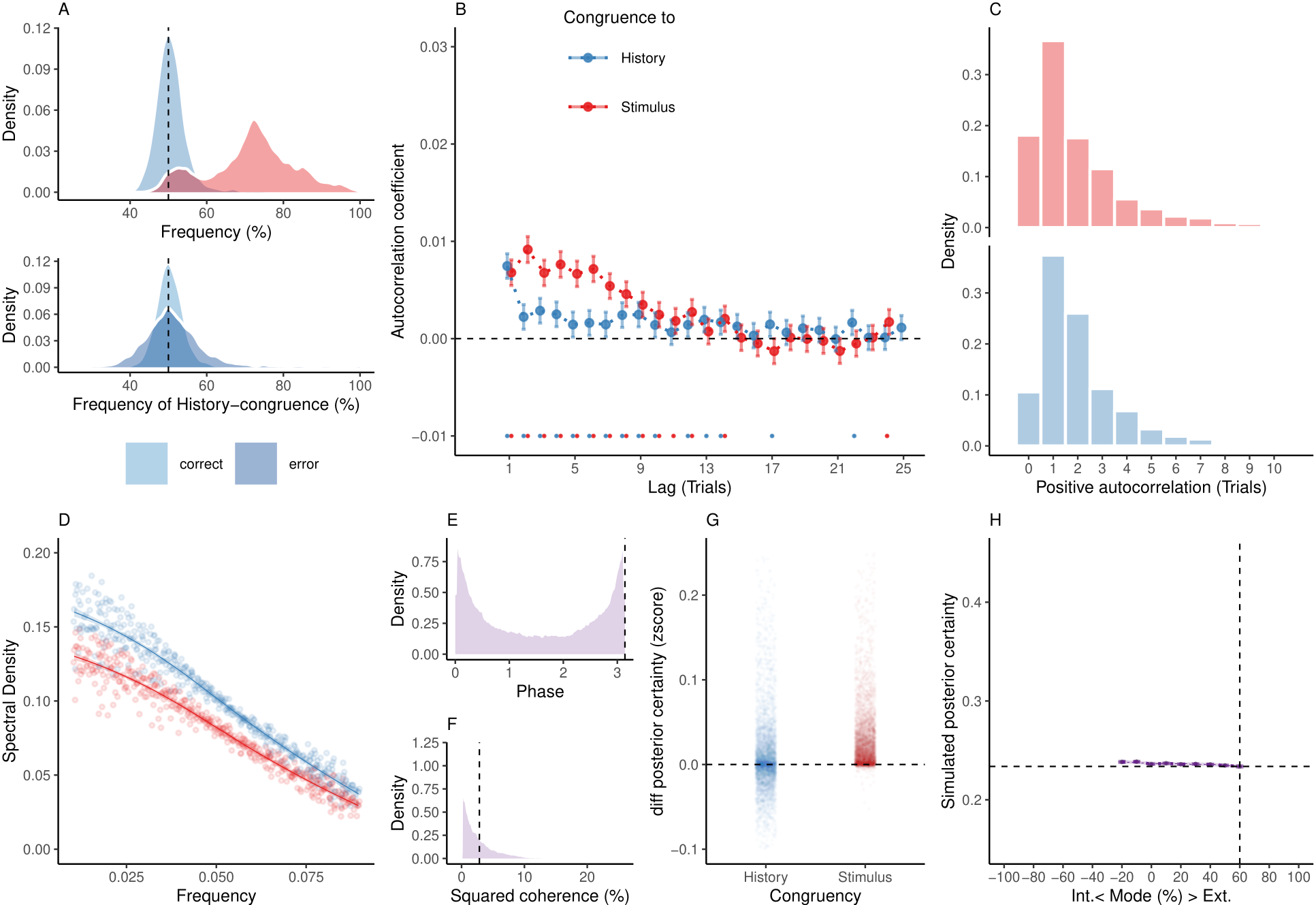

Supplement: S10 Fig — When simulating data for the likelihood-oscillation-only model, we removed the oscillation from the prior term by setting the amplitude aψ to 0. Simulated data thus depended only on the participant-wise estimates for hazard rate H, amplitude aLLR, frequency f, phase p, and inverse decision temperature ζ. (A) Similar to the full model M1 (Figs 1F and 4), simulated perceptual choices were stimulus-congruent in 71.97% ± 0.17% of trials (in red). History-congruent amounted to 50.76% ± 0.07% of trials (in blue). As in the full model, the likelihood-oscillation-only model showed a significant bias toward perceptual history T(4.32×10^3^) = 10.29, p = 1.54×10^−24^; upper panel). Similarly, history-congruent choices were more frequent at error trials (T(4.32×10^3^) = 9.71, p = 4.6×10^−22^; lower panel). (B) In the likelihood-oscillation-only model, we observed that the autocorrelation coefficients for history-congruence were reduced below the autocorrelation coefficients of stimulus-congruence. This is an approximately 5-fold reduction relative to the empirical results observed in humans (Fig 2B), where the autocorrelation of history-congruence was above the autocorrelation of stimulus-congruence. Moreover, in the reduced model shown here, the number of consecutive trials that showed significant autocorrelation of history-congruence was reduced to 11. (C) In the likelihood-oscillation-only model, the number of consecutive trials at which true autocorrelation coefficients exceeded the autocorrelation coefficients for randomly permuted data did not differ with respect to stimulus-congruence (2.62 ± 1.39×10−3 trials; T(4.32×10^3^) = 1.85, p = 0.06) but decreased with respect to history-congruence (2.4 ± 8.45×10−4 trials; T(4.32×10^3^) = −15.26, p = 3.11×10^−51^) relative to the full model. (D) In the likelihood-oscillation-only model, the smoothed probabilities of stimulus- and history-congruence (sliding windows of ±5 trials) fluctuated as a scale-invariant process with a 1/f power law, i.e [file pbio.3002410.s011.tiff]

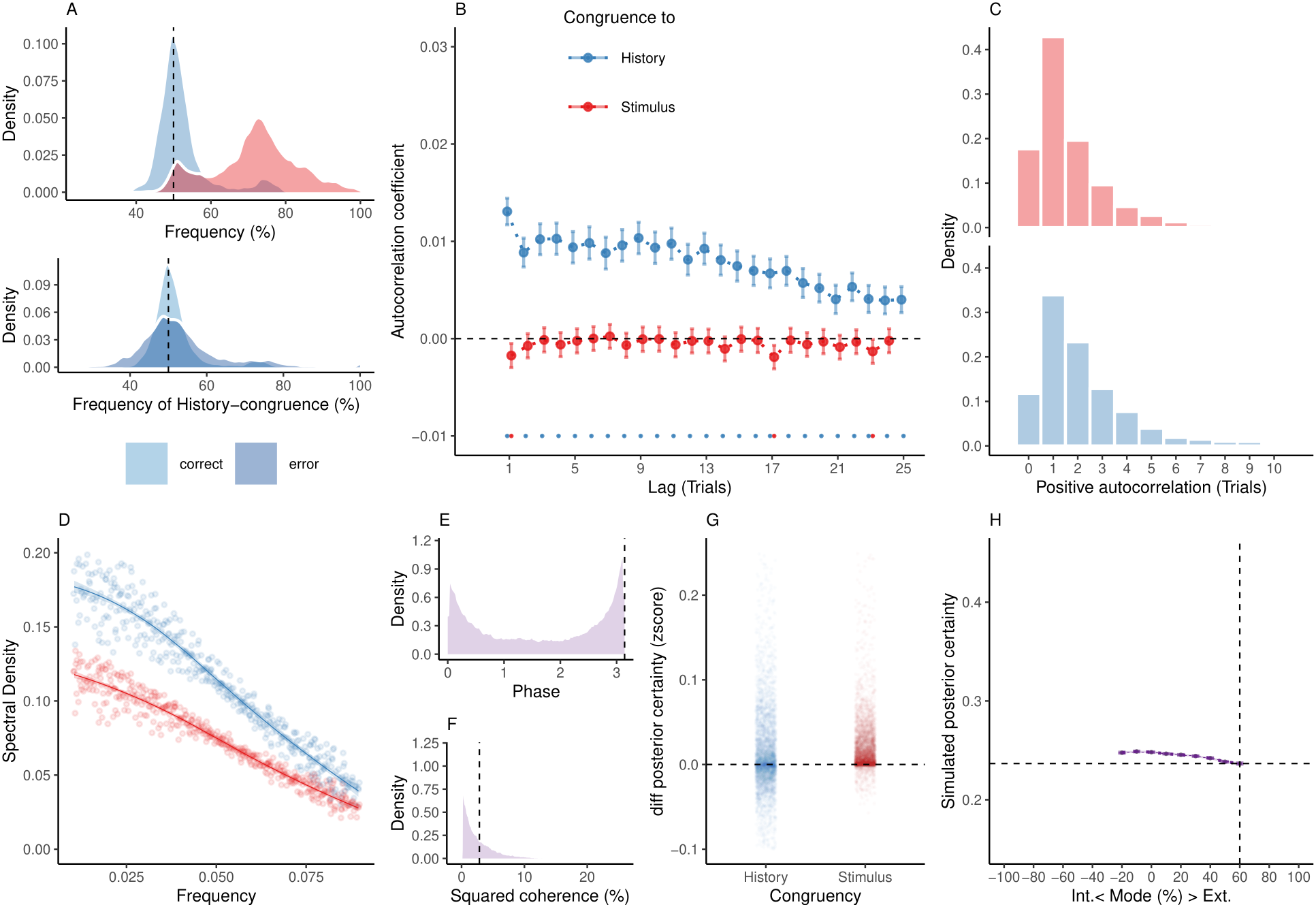

Supplement: S11 Fig — When simulating data for the prior-oscillation-only model, we removed the oscillation from the prior term by setting the amplitude aLLR to 0. Simulated data thus depended only on the participant-wise estimates for hazard rate H, amplitude aψ, frequency f, phase p, and inverse decision temperature ζ. (A) Similar to the full model (Figs 1F and 4), simulated perceptual choices were stimulus-congruent in 71.97% ± 0.17% of trials (in red). History-congruent amounted to 52.1% ± 0.11% of trials (in blue). As in the full model, the prior-oscillation-only showed a significant bias toward perceptual history T(4.32×10^3^) = 18.34, p = 1.98×10^−72^; upper panel). Similarly, history-congruent choices were more frequent at error trials (T(4.31×10^3^) = 12.35, p = 1.88×10^−34^; lower panel). (B) In the prior-oscillation-only model, we did not observe any significant positive autocorrelation of stimulus-congruence, whereas the autocorrelation of history-congruence was preserved. (C) In the prior-oscillation-only model, the number of consecutive trials at which true autocorrelation coefficients exceeded the autocorrelation coefficients for randomly permuted data did was decreased with respect to stimulus-congruence relative to the full model (1.8 ± 1.01×10−3 trials; T(4.31×10^3^) = −6.48, p = 1.03×10^−10^) but did not differ from the full model with respect to history-congruence (4.25 ± 1.84×10−3 trials; T(4.32×10^3^) = 0.07, p = 0.95). (D) In the prior-oscillation-only model, the smoothed probabilities of stimulus- and history-congruence (sliding windows of ±5 trials) fluctuated as a scale-invariant process with a 1/f power law, i.e., at power densities that were inversely proportional to the frequency (power ∼ 1/fβ; stimulus-congruence: β = −0.78 ± 1.11×10^−3^, T(1.92×10^5^) = −706.62, p < 2.2×10−308; history-congruence: β = −0.83 ± 1.27×10^−3^, T(1.92×10^5^) = −651.6, p < 2.2×10−308). (E) In the prior-oscillation-only model, the distribution of phase shift between fluctuations in simulated stimulus- a [file pbio.3002410.s012.tiff]

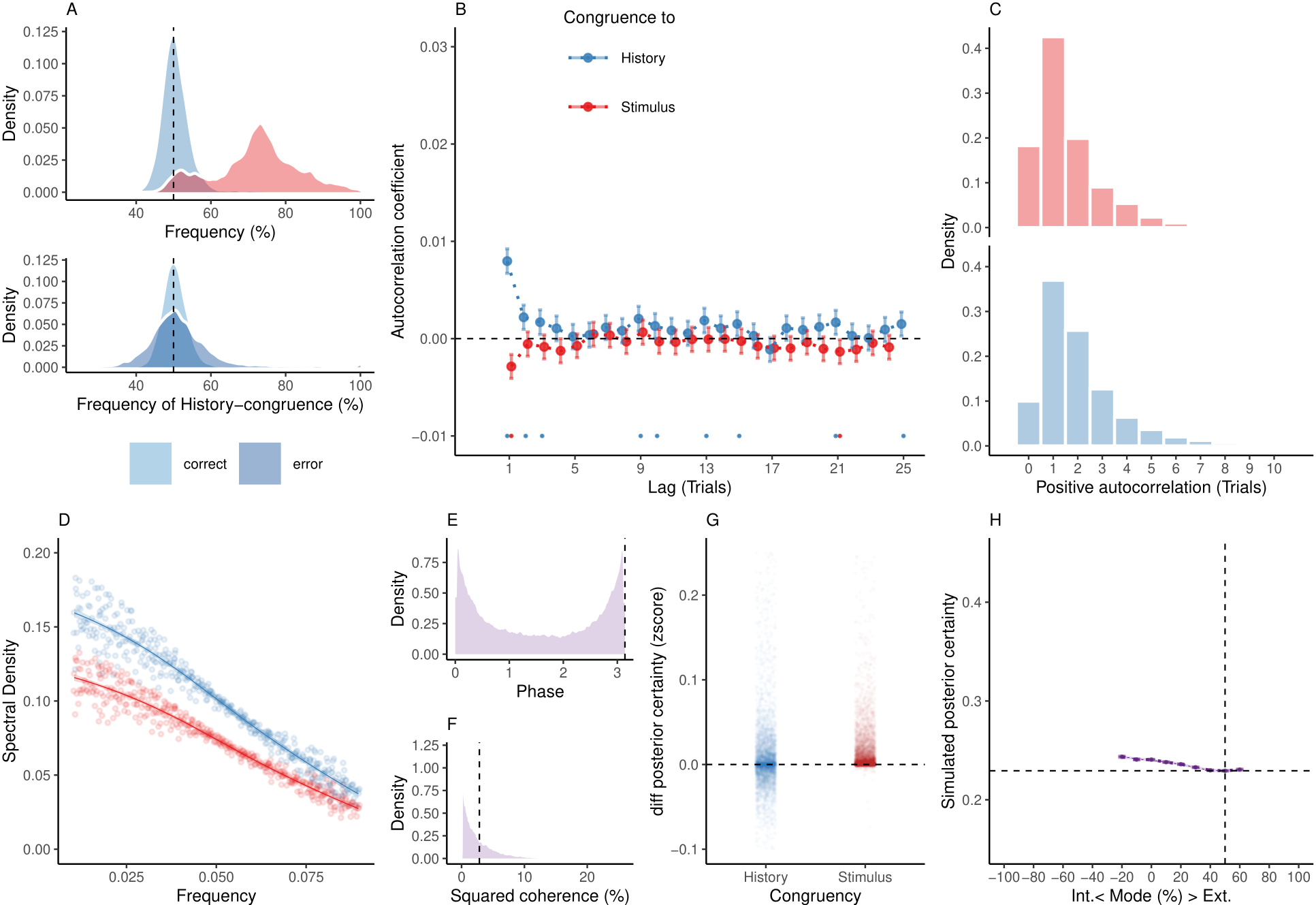

Supplement: S12 Fig — When simulating data for the normative-evidence-accumulation model, we removed the oscillation from the likelihood and prior terms by setting the amplitudes aLLR and aψ to 0. Simulated data thus depended only on the participant-wise estimates for hazard rate H and inverse decision temperature ζ. (A) Similar to the full model (Figs 1F and 4), simulated perceptual choices were stimulus-congruent in 71.97% ± 0.17% of trials (in red). History-congruent amounted to 50.73% ± 0.07% of trials (in blue). As in the full model, the no-oscillation model showed a significant bias toward perceptual history T(4.32×10^3^) = 9.94, p = 4.88×10^−23^; upper panel). Similarly, history-congruent choices were more frequent at error trials (T(4.31×10^3^) = 10.59, p = 7.02×10^−26^; lower panel). (B) In the normative-evidence-accumulation model, we did not find significant autocorrelations for stimulus-congruence. Likewise, we did not observe any autocorrelation of history-congruence beyond the first 3 consecutive trials. (C) In the normative-evidence-accumulation model, the number of consecutive trials at which true autocorrelation coefficients exceeded the autocorrelation coefficients for randomly permuted data decreased with respect to both stimulus-congruence (1.8 ± 1.59×10−3 trials; T(4.31×10^3^) = −5.21, p = 2×10^−7^) and history-congruence (2.18 ± 5.48×10−4 trials; T(4.32×10^3^) = −17.1, p = 1.75×10^−63^) relative to the full model. (D) In the normative-evidence-accumulation model, the smoothed probabilities of stimulus- and history-congruence (sliding windows of ±5 trials) fluctuated as a scale-invariant process with a 1/f power law, i.e., at power densities that were inversely proportional to the frequency (power ∼ 1/fβ; stimulus-congruence: β = −0.78 ± 1.1×10^−3^, T(1.92×10^5^) = −706.93, p < 2.2×10−308; history-congruence: β = −0.79 ± 1.12×10^−3^, T(1.92×10^5^) = −702.46, p < 2.2×10−308). (E) In the normative-evidence-accumulation model, the distribution of phase shift between fluctuations in simulated s [file pbio.3002410.s013.tiff]

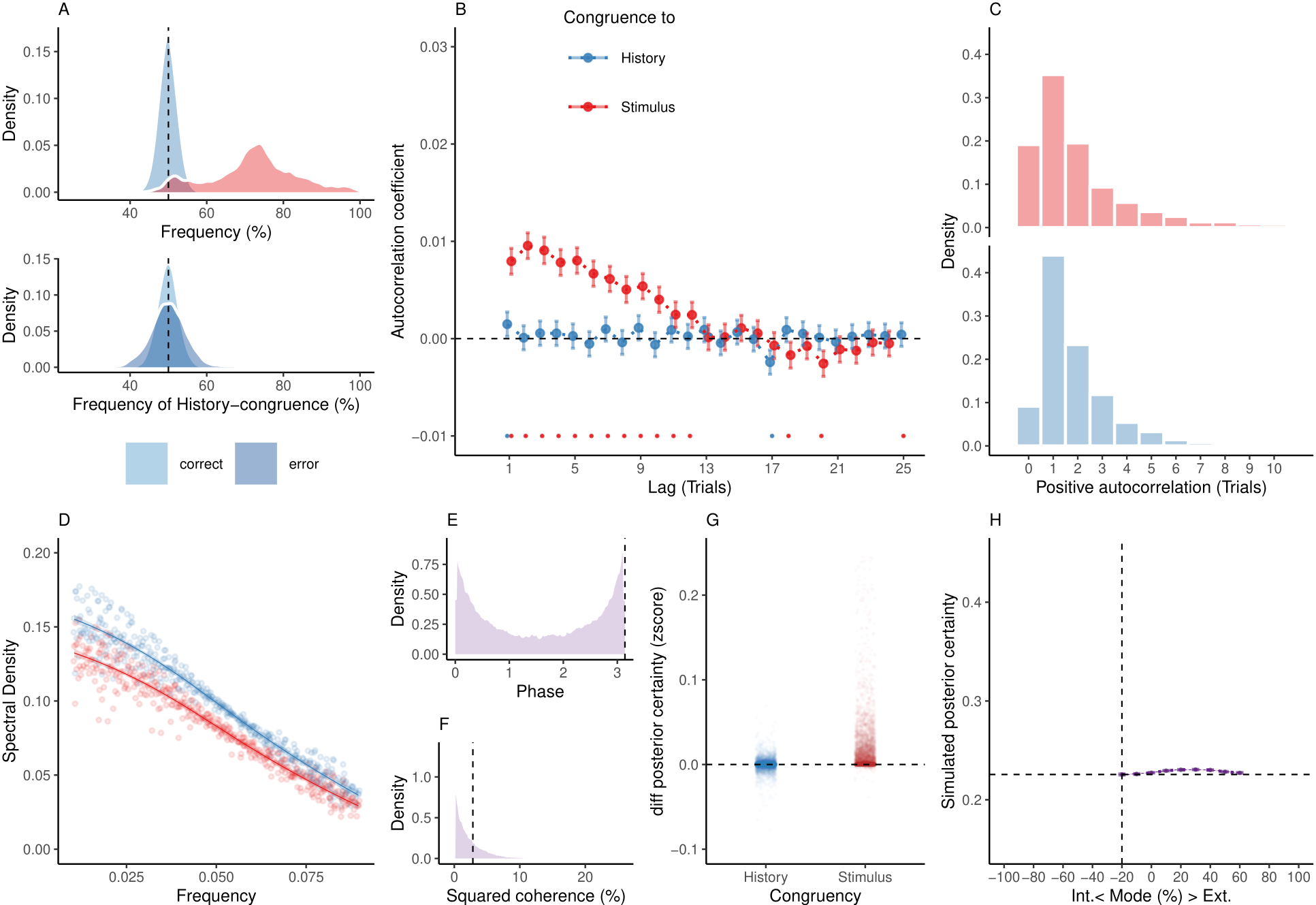

Supplement: S13 Fig — When simulating data for the no-evidence-accumulation model, we removed the accumulation of information across trials by setting the hazard rate H to 0.5. Simulated data thus depended only on the participant-wise estimates for the amplitudes aLLR/ψ, frequency f, phase p, and inverse decision temperature ζ. (A) Similar to the full model (Figs 1F and 4), simulated perceptual choices were stimulus-congruent in 72.14% ± 0.17% of trials (in red). History-congruent amounted to 49.89% ± 0.03% of trials (in blue). In contrast to the full model, the no-accumulation model showed a significant bias against perceptual history T(4.32×10^3^) = −3.28, p = 1.06×10^−3^; upper panel). In contrast to the full model, there was no difference in the frequency of history-congruent choices between correct and error trials (T(4.31×10^3^) = 0.76, p = 0.44; lower panel). (B) In the no-evidence-accumulation model, we found no significant autocorrelation of history-congruence beyond the first trial, whereas the autocorrelation of stimulus-congruence was preserved. (C) In the no-evidence-accumulation model, the number of consecutive trials at which true autocorrelation coefficients exceeded the autocorrelation coefficients for randomly permuted data increased with respect to stimulus-congruence (2.83 ± 1.49×10−3 trials; T(4.31×10^3^) = 3.45, p = 5.73×10^−4^) and decreased with respect to history-congruence (1.85 ± 3.49×10−4 trials; T(4.32×10^3^) = −19.37, p = 3.49×10^−80^) relative to the full model. (D) In the no-evidence-accumulation model, the smoothed probabilities of stimulus- and history-congruence (sliding windows of ±5 trials) fluctuated as a scale-invariant process with a 1/f power law, i.e., at power densities that were inversely proportional to the frequency (power ∼ 1/fβ; stimulus-congruence: β = −0.82 ± 1.2×10^−3^, T(1.92×10^5^) = −681.98, p < 2.2×10−308; history-congruence: β = −0.78 ± 1.11×10^−3^, T(1.92×10^5^) = −706.57, p < 2.2×10−308). (E) In the no-evidence-accumulation model, the distribution of p [file pbio.3002410.s014.tiff]

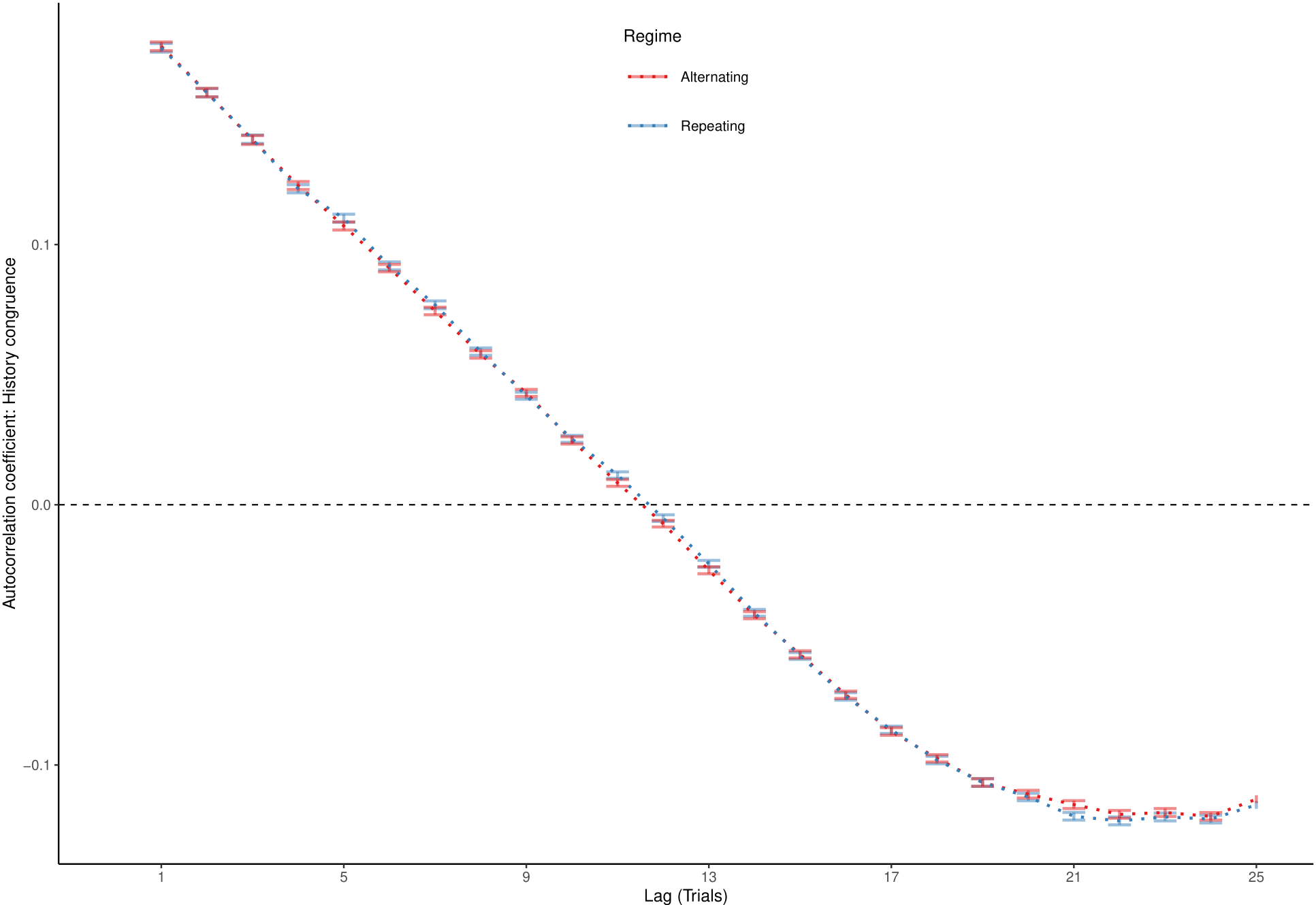

Supplement: S14 Fig — Here, we simulate the autocorrelation of history-congruence in 103 synthetic participants. In the repeating regime (blue), history-congruence fluctuated between 50% and 80% (blue) in interleaved blocks (10 blocks per condition with a random duration between 15 and 30 trials). In the alternation regime (red), history-congruence fluctuated between 50% and 20%. The resulting autocorrelation curves for history-congruence overlap, indicating that our analysis is able to accommodate both repeating and alternating biases. (TIFF) [file pbio.3002410.s015.tiff]
