# Supplementary material for: Sensory processing in humans and mice fluctuates between external and internal modes
Source: PLoS Biol. 2023 Dec 8;21(12):e3002410. doi: 10.1371/journal.pbio.3002410 (PMC10732408; doi:10.1371/journal.pbio.3002410)
Supplement: S1 Table — (PDF) [file pbio.3002410.s016.pdf]

Supplemental Table S1: Sensory processing in humans and mice fluctuates between external and internal modes

Authors:

Veith Weilnhammer[1,2,3], Heiner Stuke[1,2], Kai Standvoss[1], Philipp Sterzer[4]

Affiliations:

[1] Department of Psychiatry, Charité-Universitätsmedizin Berlin, corporate member of Freie Universität Berlin and Humboldt-Universität zu Berlin, 10117 Berlin, Germany

[2] Berlin Institute of Health, Charité-Universitätsmedizin Berlin and Max Delbrück Center, 10178 Berlin, Germany

[3] Helen Wills Neuroscience Institute, University of California Berkeley, USA

[4] Department of Psychiatry (UPK), University of Basel, Switzerland

Corresponding Author:

Veith Weilnhammer, Helen Wills Neuroscience Institute, University of California Berkeley, USA, email: veith.weilnhammer@gmail.com

Supplemental Table S1

Authors	Journal	Year
Bang, Shekhar, Rahnev	JEP:General	2019
Bang, Shekhar, Rahnev	JEP:General	2019
Calder-Travis, Charles, Bogacz, Yeung	Unpublished	NA
Clark & Merfeld	Journal of Neurophysiology	2018
Clark	Unpublished	NA
Faivre, Filevich, Solovey, Kuhn, Blanke	Journal of Neuroscience	2018
Faivre, Vuillaume, Blanke, Cleeremans	bioRxiv	2018
Filevich & Fandakova	Unplublished	NA
Gajdos, Fleming, Saez Garcia, Weindel, Davranche	Neuroscience of Consciousness	2019
Gherman & Philiastides	eLife	2018
Haddara & Rahnev	PsyArXiv	2020
Haddara & Rahnev	PsyArXiv	2020
Hainguerlot, Vergnaud, & de Gardelle	Scientific Reports	2018
Hainguerlot, Gajdos, Vergnaud, & de Gardelle	Unpublished	NA
Jachs, Blanco, Grantham-Hill, Soto	JEP:HPP	2015
Jachs, Blanco, Grantham-Hill, Soto	JEP:HPP	2015
Jachs, Blanco, Grantham-Hill, Soto	JEP:HPP	2015
Jaquiery, Yeung	Unpublished	NA
Kvam, Pleskac, Yu, Busemeyer	PNAS	2015
Kvam, Pleskac, Yu, Busemeyer	PNAS	2015
Kvam and Pleskac	Cognition	2016
Law, Lee	Unpublished	NA
Lebreton, et al.	Sci. Advances	2018
Lempert, Chen, & Fleming	PlosOne	2015
Locke*, Gaffin-Cahn*, Hosseinizaveh, Mamassian, & Landy	Attention, Perception, & Psychophysics	2020
Maniscalco, McCurdy,Odegaard, & Lau	J Neurosci	2017
Maniscalco, McCurdy,Odegaard, & Lau	J Neurosci	2017
Maniscalco, McCurdy,Odegaard, & Lau	J Neurosci	2017
Maniscalco, McCurdy,Odegaard, & Lau	J Neurosci	2017
Martin, Hsu	Unpublished	NA
Massoni & Roux	Journal of Mathematical Psychology	2017
Massoni	Unpublished	NA
Mazor, Friston & Fleming	eLife	2020
Mei, Rankine,Olafsson, Soto	bioRxiv	2019
Mei, Rankine,Olafsson, Soto	bioRxiv	2019
O'Hora, Zgonnikov, Kenny, Wong-Lin	Fechner Day proceedings	2017
O'Hora, Zgonnikov, CiChocki	Unpublished	NA

(continued)

Authors	Journal	Year
O'Hora, Zgonnikov, Neverauskaite	Unpublished	NA
Palser et al	Consciousness & Cognition	2018
Pereira, Faivre, Iturrate et al.	bioRxiv	2018
Prieto et al.	Submitted	NA
Rahnev et al	J Neurophysiol	2013
Rausch & Zehetleitner	Front Psychol	2016
Rausch et al	Attention, Perception, & Psychophysics	2018
Rausch et al	Attention, Perception, & Psychophysics	2018
Rausch, Zehetleitner, Steinhauser, & Maier	NeuroImage	2020
Recht, de Gardelle & Mamassian	Unpublished	NA
Reyes et al.	PlosOne	2015
Reyes et al.	Submitted	NA
Rouault, Seow, Gillan, Fleming	Biol. Psychiatry	2018
Rouault, Seow, Gillan, Fleming	Biol. Psychiatry	2018
Rouault, Dayan, Fleming	Nat Commun	2019
Sadeghi et al	Scientific Reports	2017
Schmidt et al.	Consc Cog	2019
Shekhar & Rahnev	J Neuroscience	2018
Shekhar & Rahnev	PsyArXiv	2020
Sherman et al	Journal of Neuroscience	2016
Sherman et al	Journal of Cognitive Neuroscience	2016
Sherman et al	Unpublished	NA
Sherman et al	Unpublished	NA
Siedlecka, Wereszczywski, Paulewicz, Wierzchon	bioRxiv	2019
Song et al	Consciousness & Cognition	2011
van Boxtel, Orchard, Tsuchiya	bioRxiv	2019
van Boxtel, Orchard, Tsuchiya	bioRxiv	2019
Wierzchon, Paulewicz, Asanowicz, Timmermans & Cleeremans	Consciousness and Cognition	2014
Wierzchon, Anzulewicz, Hobot, Paulewicz & Sackur	Consciousness and Cognition	2019
