# Supplementary material for: Sensory processing in humans and mice fluctuates between external and internal modes
Source: PLoS Biol. 2023 Dec 8;21(12):e3002410. doi: 10.1371/journal.pbio.3002410 (PMC10732408; doi:10.1371/journal.pbio.3002410)
Supplement: S2 Table — (PDF) [file pbio.3002410.s017.pdf]

Supplemental Table S2: Sensory processing in humans and mice fluctuates between external and internal modes

Authors:

Veith Weilnhammer[1,2,3], Heiner Stuke[1,2], Kai Standvoss[1], Philipp Sterzer[4]

Affiliations:

[1] Department of Psychiatry, Charité-Universitätsmedizin Berlin, corporate member of Freie Universität Berlin and Humboldt-Universität zu Berlin, 10117 Berlin, Germany

[2] Berlin Institute of Health, Charité-Universitätsmedizin Berlin and Max Delbrück Center, 10178 Berlin, Germany

[3] Helen Wills Neuroscience Institute, University of California Berkeley, USA

[4] Department of Psychiatry (UPK), University of Basel, Switzerland

Corresponding Author:

Veith Weilnhammer, Helen Wills Neuroscience Institute, University of California Berkeley, USA, email: veith.weilnhammer@gmail.com

Supplemental Table S2

Parameters	Interpretation
α	Sensitivity to sensory information
H	Expected probability of a switch in the cause of sensory information (Hazard)
a_{LLR}	Amplitude of fluctuations in likelihood precision ω_{LLR}
a_{ψ}	Amplitude of fluctuations in prior precision ω_{ψ}
f	Frequency of ω_{LLR} and ω_{ψ}
p	Phase (p for ω_{LLR}; p + π for ω_{ψ})
ζ	Inverse decision temperature
