# Supplementary material for: Sensory processing in humans and mice fluctuates between external and internal modes
Source: PLoS Biol. 2023 Dec 8;21(12):e3002410. doi: 10.1371/journal.pbio.3002410 (PMC10732408; doi:10.1371/journal.pbio.3002410)
Supplement: Attachment — Submitted filename: Response_to_Reviewers.pdf [file pbio.3002410.s018.pdf]

Response to Reviewers

Reviewer 1

This was an interesting and thought-provoking submission. I note that it is a revision: I am therefore supposing that the authors have already responded to one round of reviewer comments and that you are potentially interested in publishing this work. In brief, I think there are many elements of this report that warrant publication; however, there are some parts that are less compelling and could be deferred to a subsequent paper. The paper is far too long and would benefit greatly from being streamlined. Furthermore, some of the modeling is overengineered and is difficult to follow. I have tried to suggest how the authors might improve the presentation of their work in my comments to authors.

I enjoyed reading this long but thought-provoking report of fluctuations in the sensitivity to sensory evidence in perceptual decision-making tasks. There were some parts of this report that were compelling and interesting. Other parts were less convincing and difficult to understand. Overall, this paper is far too long. An analogy that might help here is that a dinner guest is very entertaining for the first hour or so - and then overstays their welcome; until you start wishing they would leave. Another analogy, which came to mind, was that the modeling—and its interpretation—was a bit autistic (i.e., lots of fascinating if questionable detail with a lack of central coherence).

I think that both issues could be resolved by shortening the paper and removing (or, at least, greatly simplifying) the final simulation studies of metacognition. I try to unpack this suggestion in the following.

We would like to thank Prof. Friston for the very insightful and helpful comments on our manuscript. We fully agree that our ideas about the computational function of between-mode fluctuations and the associated simulations may be presented in a more accessible form in a standalone paper. As we outlined in more detail below, we have streamlined our findings and rewrote the paper and reduced it's length by shortening the sections on computational modeling. We have also followed Prof. Friston's suggestion to interpret the effects of mode on RT and confidence in the context of predictive processing definitions of attention, namely the allocation of precision between prior and likelihood.

Major points:

As I understand it, you have used publicly available data on perceptual decision-making to demonstrate slow fluctuations in the tendency to predicate perceptual decisions on the stimuli and on the history of recent decisions. You find scale-free fluctuations in this tendency — that are anti-correlated — and interpret this as fluctuations in the precision afforded sensory evidence, relative to prior beliefs. This interpretation is based upon a model of serial dependencies (parameterised with a hazard function).

The stimulus and history (i.e., likelihood and prior) sensitivities are anti-correlated and both show scale free behavior. This is reproduced in men and mice. You then proceed to model this with periodic fluctuations in the precisions or weights applied to the likelihood and prior that are in anti-phase - and then estimate the parameters of the ensuing model. Finally, you then simulate the learning of the hazard parameter — and something called metacognition - to show that periodic fluctuations improve estimates of metacognition (based upon a Rescorla-Wagner model of learning). You motivate this by suggesting that the fluctuations in sensitivity are somehow necessary to elude circular inference and provide better estimates of precision.

Note that I am reading the parameters omega_LLR and omega_ψ as the precision of the likelihood and prior, where the precision of the likelihood is called sensory precision. This contrasts with your use of sensory precision, which seems to be attributed to a metacognitive construct M.

As noted above, all of this is fascinating but there are too many moving parts that do not fit together comfortably. I will list a few examples:

Comment 1

If, empirically, the fluctuations in sensitivity are scale-free with a 1/f power law, why did you elect to model fluctuations in precision as a periodic function with one unique timescale (i.e., f).?

The reason for choosing a unique timescale f was to enable our model to depict the dominant timescale at which prior and likelihood precision fluctuate in their impact on perceptual decision-making, giving rise to between-mode fluctuations. We think that identifying this timescale is important for planning future experiments targeted at between-mode fluctuations and their manipulation by causal interventions (e.g., pharmacology or TMS). The posterior value for f lies at approximately 0.11 $1/N_{trials}$ in both humans and mice. The value of f approximately matches the transition probabilities between *engaged* and *disengaged* states in work assessing fluctuations in perceptual decision-making using Hidden Markov models (stay probabilities ranged between 0.94 and 0.98, see Ashwood et al., Nature Neuroscience 2022). Simulating from our model (Figure 4) replicates the 1/f feature of the empirical data. Please note that the individual trial is the smallest unit of *measurement* for these fluctuations, such that our analysis is limited by definition to frequencies below 1 ($1/N_{trials}$).

We now provide a rationale for choosing one value for f - identifying the dominant timescale for fluctuations in mode - in the results section:

- To allow for *bimodal inference*, i.e., alternating periods of internally- and externally-biased modes of perceptual processing that occur irrespective of the sequence of preceding experiences, we assumed that likelihood and prior vary in their influence on the perceptual decision according to fluctuations governed by ω_{LLR} and ω_{ψ}. These anti-phase sine functions (defined by amplitudes $a_{LLR/\psi}$, frequency f and phase p) determine the precision afforded to the likelihood and prior[53]. The implicit anti-phase fluctuations are mandated by Bayes-optimal formulations in which inference

depends only on the relative values of prior and likelihood precision (i.e., the Kalman gain[54]). As such, ω_{LLR} and ω_{ψ} implement a hyperprior[55] in which the likelihood and prior precisions are shifted against each other at a dominant timescale defined by f: (...)

- (...) The parameter f captures the dominant time scale at which likelihood (amplitude humans: a_{LLR} = 0.5 ± 2.02 × 10^{-4}; mice: a_{LLR} = 0.39 ± 1.08 × 10^{-3} and prior precision (amplitude humans: a_{ψ} = 1.44 ± 5.27 × 10^{-4}; mice: a_{ψ} = 1.71 ± 7.15 × 10^{-3} were shifted against each other and was estimated at 0.11 ± 1.68 × 10^{-5} $1/N_{trials}$ and 0.11 ± 1.63 × 10^{-4} $1/N_{trials}$ in mice.
- Table 2 (see response to Comment 22 by Reviewer 1) contains an additional definition for all model parameters, including f.

Comment 2

At present, the estimates of meta-cognition (M) play the role of accumulated estimates of (sensory or prior) precision. Why are these not used in your model of perceptual decisions in Equation 2.

In our model, the parameter α controls the encoding precision by governing the transformation from sensory stimuli to the log likelihood ratio (LLR) via the equations (13-16) (the LLR ends up closer to zero when α is low). Our simulations on the adaptive benefits of bimodal inference rest on the assumption that α may change unpredictably. The construct M is a belief about α that may be useful for, e.g., communicating the precision of sensory encoding to other cognitive domains or agents. To our mind, α is a feature of low-level sensory encoding that cannot be modulated by top-down beliefs such as M. This is why we did not include M in equation (2). Please note that we have removed this section following Comment 9 of Reviewer 1.

Comment 3

Why do you assume that non-specific increases in attention and arousal will increase reaction times? If one has very precise prior beliefs (and is not attending to stimuli), would you not expect a decrease in reaction time?

Thanks a lot for pointing this out (see also the Comment below and Comment 6 by Reviewer 3). Both high prior and high likelihood precision lead to higher absolute values of the log posterior ratio (reflecting certainty encoded by the decision variable), and thus faster RTs. This is reflected empirically by RTs in humans (Figure 2) and to a lesser degree in mice (Figure 3): RTs tended to be shorter for stronger biases toward both external and internal mode. Our model, which incorporates (i), the accumulation of information across trials, and (ii), fluctuations in the likelihood precision relative to the prior precision, recapitulates this feature of the data, which is lost or greatly attenuated when eliminating process (i) and/or (ii) (see model comparison and simulations below). Our data thus confirm the hypothesis that both high prior and likelihood precision lead to faster RTs.

In the previous version of the manuscript, we had included the relation between mode and RTs and confidence primarily as a defensive analysis against the proposition that what we call between-mode fluctuations is not a perceptual phenomenon, but occurs downstream of perception. One may imagine that fluctuations in perceptual performance are not influenced by periods of relative increases in prior precision (which decrease performance in fully randomized designs), but by periods when participants do not attend to the task at all, i.e., neither to sensory information nor to prior precision. We propose that the analyses of RTs and confidence can give some insight into whether such alternative mechanisms may be at play, as we would assume longer RTs and lower confidence if participants failed to attend to the task at all.

That being said, we realize that, due to the potential non-linearity in their relation to arousal (see also Comment 6 by Reviewer 3), RTs and confidence cannot provide a definitive map of where fluctuations in mode are situated in relation to arousal. Such a delineation may potentially be provided by tracking of pupil size, response behavior or by neural data (e.g., noise correlations of fluctuations in LFP). These data are not available for the studies in the Confidence Database, but were very recently published for the IBL database (after this manuscript was submitted). While we believe that this is beyond the scope of this manuscript, we will assess the relation of pupil diameter, motor behavior (turning of the response wheel) and LFPs to between-mode fluctuations in a future publication using the IBL dataset.

In light of the considerations above and our response to Comment 6 by Reviewer 3, we now refer to attention in the predictive processing sense. We use the term "task engagement" instead of "on-task attention" to refer to situation in which participants may not attend to the task at all, e.g. due to low arousal or fatigue, and discuss these as alternative explanations for between-mode fluctuations. We have made three sets of changes to our manuscript:

First, we present our results on the relation of mode to RT and Confidence in a more descriptive way, and do not use it as a strong defensive analysis against arousal:

- The above results point to systematic fluctuations in the *decision variable*[44] that determines perceptual choices, causing enhanced sensitivity to external stimulus information during external mode and increased biases toward preceding choices during internal mode. As such, fluctuations in mode should influence downstream aspects of behavior and cognition that operate on the perceptual decision variable[44]. To test this hypothesis with respect to motor behavior and metacognition, we asked how bimodal inference relates to response times (RTs) and confidence reports. (....)

- (…) In sum, the above results indicate that reporting behavior and metacognition do not map linearly onto the mode of sensory processing. Rather, they suggest that slow fluctuations in the respective impact of external and internal information are most likely to affect perception at an early level of sensory analysis[46,47]. Such low-level processing may thus integrate perceptual history with external inputs into a decision variable[44] that influences not only perceptual choices, but also the speed and confidence at which they are made.

- In what follows, we probe alternative explanations for between-mode fluctuations, test for the existence of modes in mice, and propose a predictive processing model that explains fluctuations in mode by ongoing shifts in the precision afforded to external sensory information relative to internal predictions driven by perceptual history.

Second, we have re-written our discussion of the quadratic relationship of mode to RTs and Confidence, focusing on predictive processing models of attention: - As a functional explanation for bimodal inference, we propose that perception temporarily disengages from internal predictions to form stable inferences about the statistical properties of the sensory environment. Between-mode fluctuations may thus elude circular inferences that occur when both the causes and the encoding of sensory stimuli are volatile[19,57].** By the same token, we suggest that fluctuations in mode occur at the level of perceptual processing[26,30,46,47], and are not a passive phenomenon that is primarily driven by factors situated up- or downstream of sensory analysis.

- How does attention relate to between-mode fluctuations? According to predictive processing, attention corresponds to the precision afforded to the probability distributions that underlie perceptual inference[53]. From this perspective, fluctuations between external and internal mode can be understood as ongoing shifts in the attention afforded to either external sensory information (regulated via likelihood precision) or internal predictions (regulated via prior precision). When the precision of either likelihood or prior increases, posterior precision increases, which leads to faster RTs and higher confidence. Therefore, when defined from the perspective of predictive processing as the precision afforded to likelihood and prior[53], fluctuations in attention may provide a plausible explanation for the quadratic relationship of mode to RTs and confidence (Figure 2H and J; Figure 3I; Figure 3I).

Third, we have added a more general discussion of our findings in the light of fluctuations in task engagement:

- Outside of the predictive processing field, attention is often understood in the context of task engagement[63], which varies according to the availability of cognitive resources that are modulated by factors such as tonic arousal, familiarity with the task, or fatigue[63]. Our results suggest that internal mode processing cannot be completely reduced to intervals of low task engagement: In addition to shorter RTs and elevated confidence, choices during internal mode were not random or globally biased, but driven by perceptual history (Supplemental Figures S6-7). Moreover, our computational model identified the dominant timescale of between-mode fluctuations at 0.11 $1/N_{trials}$, which may be compatible with fluctuations in arousal[64], but is faster than to be expected for the development of task familiarity or fatigue.

- However, in interpreting the impact of between-mode fluctuations on perceptual accuracy, speed of response and confidence, it is important to consider that global modulators such as tonic arousal are known to have non-linear effects on task performance[65]: In perceptual tasks, performance seems so be highest during mid-level arousal, whereas low- and high-level arousal lead to reduced accuracy and

slower responses[65]. This contrasts with the effects of bimodal inference, where accuracy increases linearly as one moves from internal to external mode, and responses become faster at both ends of the mode spectrum.

- Of note, high phasic arousal has been shown to suppress multi-domain biases in decision-making in humans and mice[66–68], including the biases toward perceptual history[28] that we implicate in internal mode processing. The increase in response speed and history congruence over time (Supplemental Section 1.4) may argue against insufficient training as an alternative explanation for internal mode processing, but may also taken as a sign of waning arousal. The multiple mechanistic mappings to RTs and confidence therefore warrant more direct measures of arousal (such as pupil size[28,65,66,68–70], motor behavior[69,70], or neural data[71]) to better delineate bimodal inference from fluctuations in global modulators of task performance.

Comment 4

In the predictive processing literature, attention is thought to correspond to fluctuations in sensory and prior precision. Why did you then consider attention as some additional or unrelated confound?

This point is closely related to the comment above. We realize that, in the predictive processing field, attention is equated with the precision of probability distributions that contribute to the perceptual decision, such that an observer can attend strongly to sensory information (high likelihood precision) or to internal predictions derived from the sequence of preceding percepts (high prior precision). Therefore, when following the above predictive processing definition, fluctuations in attention can be equated with fluctuations in mode.

However, we feel that outside of the predictive processing field, attention is not always conceived in that way, such that low attention is often considered as low engagement with the task, i.e., relating to low likelihood and low prior precision in the predictive coding sense. It is against these caveats that our control analysis (based on psychometric functions, training effects, RTs and confidence) defend our conclusions on bimodal inference.

We now provide a more nuanced interpretation of our findings of RTs and confidence in relation to attention, with a specific focus on predictive coding and precision. We hope that our responses to the comment above resolve the points raised in this comment.

Comment 5

What licences the assumption that "agents depend upon internal confidence signals" in the absence of feedback?

In the absence of feedback, observers can only rely on internal estimates of performance to guide updates to their model of the reliability of their sensory apparatus (inferences about M). Previous work (e.g. Guggenmos et al., Elife 2106, *https://doi.org/10.7554/eLife.13388*) has shown that confidence signals can provide signals that drive perceptual learning in the absence of feedback. This has motivated our model simulation on the adaptive benefits of bimodal inference for metacognition, where the learning signal ϵ_M (i.e., the difference

between the choice and its probability) drives inferences about M. Please note that we have removed this section following Comment 9 of Reviewer 1.

Comment 6

And what licences the assumption that internal confidence feedback corresponds to "the absolute of the posterior log ratio" (did you mean the log of the posterior ratio)?

We mean the absolute of the log of the posterior ratio. Following first order models (see e.g., Fleming & Daw, Self-evaluation of decision-making: A general Bayesian framework for metacognitive computation, Psychol. Rev. 2017, *https://doi.org/10.1037/rev0000045*), the perceptual decision and the confidence report rely on the posterior. The distance of the log of the posterior ratio L_t from zero becomes a measure of decision-certainty or confidence.

Comment 7

I got a bit lost here when you say that "the precision of sensory coding M a function of u_t. This is largely because I couldn't find a definition of u_t.

We apologize for this lack of clarity. In the model simulations on the adaptive benefits of bimodal inference, we generated stimuli s_t from a Bernoulli-distribution with p = q = 0.5. The value of u_t was then defined via equation (13), following our modeling of the human data. Please note that we have removed this section following Comment 9 of Reviewer 1.

Comment 8

What licences an application of Rescorla-Wagner to learning the parameters (as in Equation 11) and, learning sensory precision as described by M_T (Equation 13). Are you moving from a Bayesian framework to a reinforcement learning framework?

We would like to thank the reviewer for pointing out this inconsistency. We have chosen the Rescorla-Wagner learning rule for simplicity: In our model, the speed of learning about H and M varied according to the current mode of perceptual processing and a constant learning rate. Allowing the learning rate itself to vary as a function of preceding experiences would add an additional level of complexity that we sought to omit in this analysis. However, we fully agree that choosing a Bayesian framework (e.g., a three-level HGF) would indeed be more consistent. Please note that we have removed this section following Comment 9 of Reviewer 1.

Comment 9

I am sure you have answers to these questions - but with each new question the reader is left more and more skeptical that there is a coherent story behind your analyses. It would have been more convincing had you just committed to a Bayesian filter and made your points using one update scheme, under ideal Bayesian observer assumptions.

Unlike your piecemeal scheme, things like the hierarchical Gaussian filter estimates the sensory and prior decisions explicitly and these estimates underwrite posterior inference. In your scheme, the sensory precision M appears to have no influence on

perceptual inference (which is why, presumably you call it metacognition). The problem with this is that your motivation for systematic fluctuations in precision is weakened. This is because improved metacognition does not improve perception — it only improves the perception of perception.

In light of the above, can I suggest that you remove Section 5.8 and use your model in the preceding section to endorse your hypothesis along the following lines:

"In summary, we hypothesized that subjects have certain hyperpriors that are apt for accommodating fluctuations in the predictability of their environment; i.e., people believe that their world is inherently volatile. This means that to be Bayes optimal it is necessary to periodically re-evaluate posterior beliefs about model parameters. One way to do this is to periodically suspend the precision of prior beliefs and increase the precision afforded to sensory evidence that updates (Bayesian) beliefs about model parameters. The empirical evidence above suggests that the timescale of this periodic scheduling of evidence accumulation may be scale-invariant. This means that there may exist a timescale of periodic fluctuations in precision over every window or length of perceptual decision-making. In what follows, we model perceptual decisions under a generative model (based upon a hazard function to model historical or serial dependencies) with, a periodic fluctuation in the precision of sensory evidence relative to prior beliefs at a particular timescale. Remarkably—using Bayesian model comparison—we find that a model with fluctuating precisions has much greater evidence, relative to a model in the absence of fluctuating precisions. Furthermore, we were able to quantify the dominant timescale of periodic fluctuations; appropriate for these kinds of paradigm."

Note, again, I am reading your omega_LLR and omega_ψ as precisions and that the periodic modulation is the hyperprior that you are characterizing—and have discovered.

We would like to thank Prof. Friston for these very helpful and precise suggestions. In brief, we now provide a quantitative assessment of model space based on AIC (i) and have followed the suggestion of omitting section 5.8 (ii).

In addition to the qualitative assessment of our models in the initial version of our manuscript, we have conducted a formal model comparison (i). Following the model comparisons in other sections of the manuscript, we based the model comparison on AIC. We furthermore show that the winning *bimodal inference model* predicts out-of-training variables (RT and confidence) and use simulations from posterior model parameters to show that, in contrast to reduced models, the bimodal inference model neither over- nor underfits the empirical data. We have added a description of our model comparison to the Method section:

- We validated the bimodal inference model in three steps: a formal model comparison to reduced models based on AIC (Figure 1F-G; Supplemental Figure S9), the prediction of within-training (stimulus- and history-congruence) as well as out-of-training variables (RT and confidence), and a qualitative reproduction of the empirical data from model simulations based on estimated parameters (Figure 4).

- **Model comparison**. We assessed the following model space based on AIC:

- The full *bimodal inference model* (M1; Figure 1F) incorporates the influence of sensory information according to the parameter α (likelihood); the integration of evidence across trials according to the parameter H (prior); anti-phase oscillations in between likelihood and prior precision according to ω_{LLR} and ω_{ψ} with parameters a_{LLR} (amplitude likelihood fluctuation), a_{ψ} (amplitude prior fluctuation), f (frequency) and p (phase).

- The *likelihood-oscillation-only model* (M2; Figure 1G) incorporates the influence of sensory information according to parameter α (likelihood); the integration of evidence across trials according to parameter H (prior); oscillations in likelihood precision according to ω_{LLR} with parameters a_{LLR} (amplitude likelihood fluctuation), f (frequency) and p (phase).

- The *prior-oscillation-only model* (M3; Figure 1G) incorporates the influence of sensory information according to parameter α (likelihood); the integration of evidence across trials according to parameter H (prior); oscillations in the prior precision according to ω_{ψ} with parameters a_{ψ} (amplitude prior fluctuation), f (frequency) and p (phase). Please note that all models M1-3 lead to shifts in the relative precision of likelihood and prior.

- The *normative-evidence-accumulation* (M4; Figure 1G) incorporates the influence of sensory information according to parameter α (likelihood); the integration of evidence across trials according to parameter H (prior), There are no additional oscillations. Model M4 thus corresponds to the model proposed by Glaze et al. and captures normative evidence accumulation in unpredictable environments using a Bayesian update scheme[51]. The comparison against M4 tests the null hypothesis that fluctuations in mode emerge from a normative Bayesian model without the ad-hoc addition of oscillations as in models M1-3.

- The *no-evidence-accumulation* (M5; Figure 1G) incorporates the influence of sensory information according to parameter α (likelihood). The model lacks integration of evidence across trials (flat prior) and oscillations. The comparison against M5 tests the null hypothesis that observers do not use prior information derived from serial dependency in perception.

- **Prediction of within-training and out-of-training variables**. To validate our model, we correlated individual posterior parameter estimates with the respective conventional variables. As a sanity check, we tested (i), whether the estimated hazard rate H correlated negatively with the frequency of history-congruent choices and, (ii), whether the estimated sensitivity to sensory information α correlated positively with the frequency of stimulus-congruent choices. In addition, we tested whether the posterior decision certainty (i.e.. the absolute of the log posterior ratio) correlated negatively with RTs and positively with confidence. This allowed us to assess whether our model could explain aspects of the data it was not fitted to (i.e., RTs and confidence).

- **Simulations**. Finally, we used simulations (Figure 4, Supplemental Figures S10-13) to show that all model components, including the anti-phase oscillations governed by a_ψ, a_{LLR}, f and p, were necessary for our model to reproduce the characteristics of the empirical data. This enabled us to assess over- or under-fitting in the bimodal inference model and all reduced models M2-M5. We used the posterior model parameters observed for humans (H, α, a_ψ, a_{LLR}, f and ζ) to define individual parameters for simulation in 4317 simulated participants (i.e., equivalent to the number of human participants). For each participant, the number of simulated trials was drawn at random between 300 to 700. Inputs s were drawn at random for each trial, such that the sequence of inputs to the simulation did not contain any systematic seriality. Noisy observations u were generated by applying the posterior parameter α to inputs s, thus generating stimulus-congruent choices in $71.36 \pm 2.6 \times 10^{-3}\%$ of trials. Choices were simulated based on the trial-wise choice probabilities y_p obtained from our model. Simulated data were analyzed in analogy to the human and mouse data. As a substitute of subjective confidence, we computed the absolute of the trial-wise log posterior ratio $|L|$ (i.e., the posterior decision certainty).

We have also added a graphical depiction of the model space to Figure 1 (subpanels F and G):

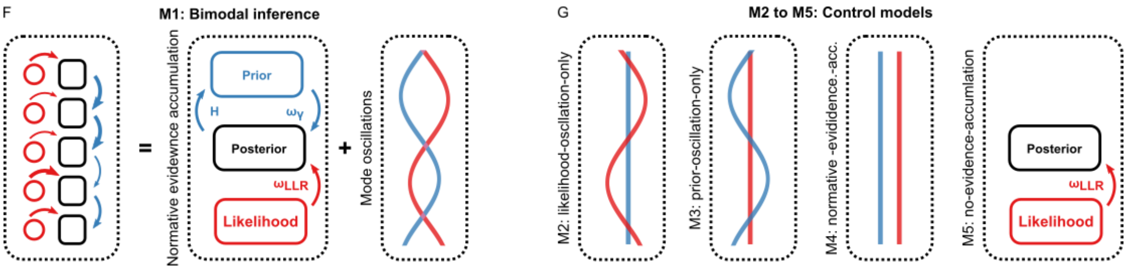

- F. The bimodal inference model (M1) explains fluctuations between externally- and externally-biased modes (left panel) by two interacting factors: a normative accumulation of evidence according to parameters H (middle panel), and anti-phase oscillations in the precision terms ω_{LLR} and ω_ψ (right panel).

- G. The control models M2-M5 were constructed by successively removing the anti-phase oscillations and the integration of information from the bimodal inference model. Please note that the normative-evidence-accumulation-model (M4) corresponds to the model proposed by Glaze et al.[51]. In the no-evidence-accumulation model (M5), perceptual decisions depend only on likelihood information (flat priors).

The formal model comparison yielded clear evidence for a superiority of the bimodal inference model, in particular over the normative Bayesian model of evidence accumulation. The model successfully predicted both within-training variables (as a sanity-check) and out-of-training variables. Simulations from posterior model parameters closely followed the empirical data (Figure 4), which was not the case for reduced models (Supplemental Figures S10-13). We summarize these findings in the Results section and have added a Supplemental Figure S9 to show the distribution of observer-level AIC at the session-level (see below):

- We used a maximum likelihood procedure to fit the bimodal inference model (M1, Figure 1F) to the behavioral data from the Confidence database[20] and the IBL database[21], optimizing the parameters α, H, amp_{LLR}, amp_{ψ}, f, p and ζ (see Methods for details and Supplemental Table T2 for a summary of the parameters of the bimodal inference model). We validated our model in three steps: First, to show that bimodal inference does not emerge spontaneously in normative Bayesian models of evidence accumulation, but requires the ad-hoc addition of anti-phase oscillations in prior and likelihood precision, we compared the bimodal inference model to four control models (M2-5, Figure 1G). In these models, we successively removed the anti-phase oscillations (M2-M4) and the integration of information across trials (M5) from the bimodal inference model and performed a model comparison based on AIC.

- Model M2 (AIC_2 = 5.7×10^4 in humans and 4.94×10^4 in mice) and Model M3 (AIC_3 = 6.9×10^4 in humans and 5.99×10^4 in mice) incorporated only oscillations of either likelihood or prior precision. Model M4 (AIC_4 = 9.8×10^4 in humans and 9.19×10^4 in mice) lacked any oscillations of likelihood and prior precision and corresponded to the normative model proposed by Glaze et al.[51]. In model M5 (AIC_4 = 1.16×10^5 in humans and 1.14×10^5 in mice), we furthermore removed the integration of information across trials, such that perception depended only in incoming sensory information (Figure 1G).

- The bimodal inference model achieved the lowest AIC across the full model space (AIC_1 = 4.73×10^4 in humans and 4.28×10^4 in mice) and was clearly superior to the normative Bayesian model of evidence accumulation (δ_{AIC} = -5.08×10^4 in humans and -4.91×10^4 in mice; Supplemental Figure S9).

- As a second validation of the bimodal inference model, we tested whether the posterior model predicted within-training and out-of-training variables. The bimodal inference model characterizes each subject by a sensitivity parameter α (humans: α = 0.5 ± 1.12×10^{-4}; mice: α = 1.06 ± 2.88×10^{-3}) that captures how strongly perception is driven by the available sensory information, and a hazard rate parameter H (humans: H = 0.45 ± 4.8×10^{-5}; mice: H = 0.46 ± 2.97×10^{-4}) that controls how heavily perception is biased by perceptual history. The parameter f captures the dominant time scale at which likelihood (amplitude humans: a_{LLR} = 0.5 ± 2.02×10^{-4}; mice: a_{LLR} = 0.39 ± 1.08×10^{-3}) and prior precision (amplitude humans: a_{ψ} = 1.44 ± 5.27×10^{-4}; mice: a_{ψ} = 1.71 ± 7.15×10^{-3}) fluctuated and was estimated at 0.11 ± 1.68×10^{-5} $1/N_{trials}$ and 0.11 ± 1.63×10^{-4} $1/N_{trials}$ in mice.

- As a sanity check for model fit, we tested whether the frequency of stimulus- and history-congruent trials in the Confidence database[20] and IBL database[21] correlate with the estimated parameters α and H, respectively. As expected, the estimated sensitivity toward stimulus information α was positively correlated with the frequency of stimulus-congruent perceptual choices (humans: β = 8.4 ± 0.26, T(4.31×10^3) = 32.87, p = 1.3×10^{-211}; mice: β = 1.93 ± 0.12, T(2.07×10^3) = 16.21, p = 9.37×10^{-56}). Likewise, H was negatively correlated with the frequency of history-congruent perceptual choices (humans: β = -11.84 ± 0.5, T(4.29×10^3) =

-23.5, p = 5.16×10^{-115}; mice: β = -6.18 ± 0.66, $T(2.08 \times 10^{3})$ = -9.37, p = 1.85×10^{-20}).

- Our behavioral analyses reveal that humans and mice show significant effects of perceptual history that impaired performance in randomized psychophysical experiments[24,28,30,31,43] (Figure 2A and 3A). We therefore expected that humans and mice underestimated the true hazard rate $\widehat{H}$ of the experimental environments (Confidence database[20]: $\widehat{H}_{Humans}$ = 0.5 ± 1.58^{-5}); IBL database[21]: $\widehat{H}_{Mice}$ = 0.49 ± 6.47^{-5}). Indeed, when fitting the bimodal inference model to the trial-wise perceptual choices, we found that the estimated (i.e., subjective) hazard rate H was lower than $\widehat{H}$ for both humans ($\beta = -6.87 \pm 0.94$, $T(61.87) = -7.33$, p = 5.76×10^{-10}) and mice ($\beta = -2.91 \pm 0.34$, $T(112.57) = -8.51$, p = 8.65×10^{-14}).

- To further probe the validity of the bimodal inference model, we tested whether posterior model quantities could explain aspects of the behavioral data that the model was not fitted to. We predicted that the posterior decision variable L_t not only encodes perceptual choices (i.e., the variable used for model estimation), but should also predict the speed of response and subjective confidence[30,44]. Indeed, the estimated trial-wise posterior decision certainty $|L_t|$ correlated negatively with RTs in humans ($\beta = -4.36 \times 10^{-3} \pm 4.64 \times 10^{-4}$, $T(1.98 \times 10^{6}) = -9.41$, p = 5.19×10^{-21}) and TDs mice ($\beta = -35.45 \pm 0.86$, $T(1.28 \times 10^{6}) = -41.13$, p = 0). Likewise, subjective confidence reports were positively correlated with the estimated posterior decision certainty in humans (β = $7.63 \times 10^{-3} \pm 8.32 \times 10^{-4}$, $T(2.06 \times 10^{6})$ = 9.18, p = 4.48×10^{-20}).

- The dynamic accumulation of information inherent to our model entails that biases toward perceptual history are stronger when the posterior decision certainty at the preceding trial is high[30,31,51]. Due to the link between posterior decision certainty and confidence, we reasoned that confident perceptual choices should be more likely to induce history-congruent perception at the subsequent trial[30,31]. Indeed, logistic regression indicated that history-congruence was predicted by the posterior decision certainty $|L_{t-1}|$ (humans: $\beta = 8.22 \times 10^{-3} \pm 1.94 \times 10^{-3}$, z = 4.25, p = 2.17×10^{-5}; mice: $\beta = -3.72 \times 10^{-3} \pm 1.83 \times 10^{-3}$, z = -2.03, p = 0.04) and subjective confidence (humans: $\beta = 0.04 \pm 1.62 \times 10^{-3}$, z = 27.21, p = 4.56×10^{-163}) at the preceding trial.

- As a third validation of the bimodal inference model, we used the posterior model parameters to simulate synthetic perceptual choices and repeated the behavioral analyses conducted for the empirical data. Simulations from the bimodal inference model closely replicated our empirical results: Simulated perceptual decisions resulted from a competition of perceptual history with incoming sensory signals (Figure 4A). Stimulus- and history-congruence were significantly autocorrelated (Figure 4B-C), fluctuating in anti-phase as a scale-invariant process with a 1/f power law (Figure 4D-F). Simulated posterior certainty[28,30,44] (i.e., the absolute of the log posterior ratio $|L_t|$) showed a quadratic relationship to the mode of sensory processing (Figure 4H), mirroring the relation of RTs and confidence reports to external and internal biases in perception (Figure 2G-H and Figure 3G-H). Crucially,

the overlap between empirical and simulated data broke down when we removed the anti-phase oscillations or the accumulation of evidence over time from the bimodal inference model (Supplemental Figure S10-13).

- In sum, computational modeling suggested that between-mode fluctuations are best explained by two interlinked processes (Figure 1E): (i), the dynamic accumulation of information across successive trials mandated by normative Bayesians model of evidence accumulation and, (ii), ongoing anti-phase oscillations in the impact of external and internal information.

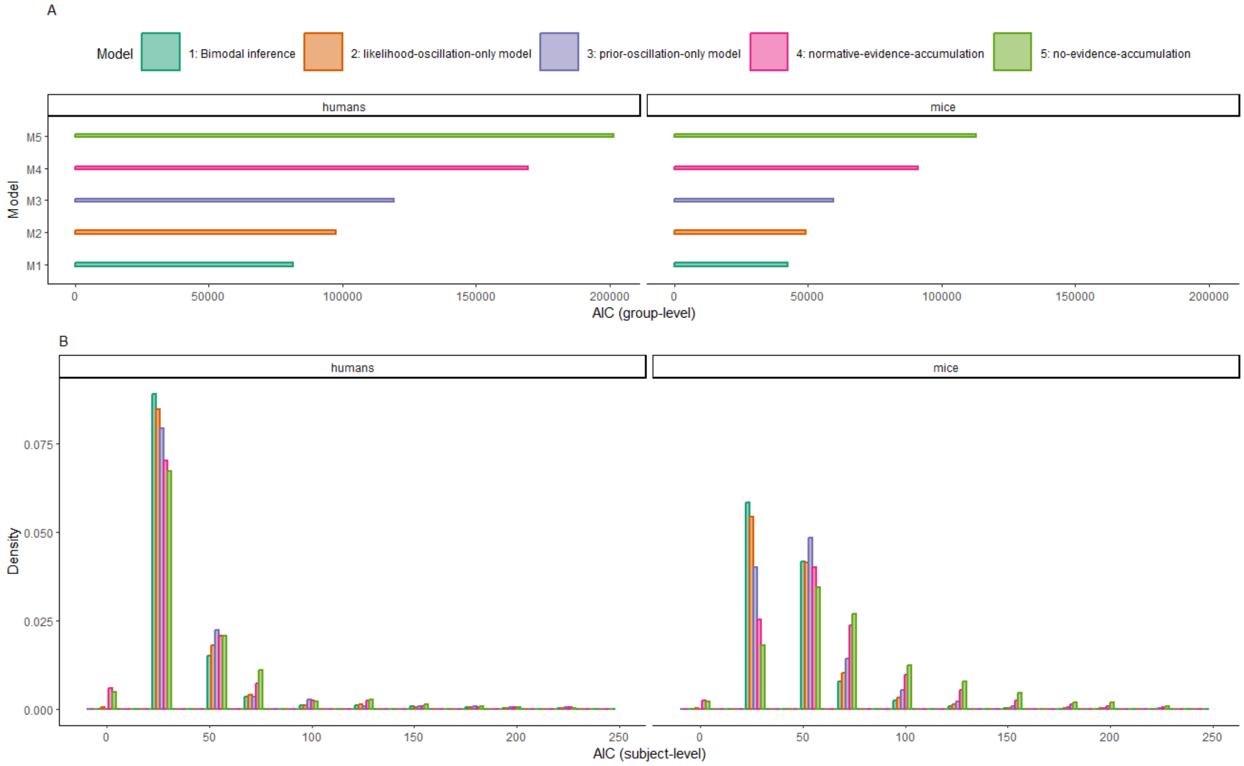

- **Supplemental Figure S9**. Comparison of the bimodal inference model against reduced control models.

- A. Group-level AIC. The bimodal inference model (M1) achieved the lowest AIC across the full model space (AIC_1 = 4.73^{4} in humans and 4.28^{4} in mice). Model M2 ($AIC_2 = 5.7 \times 10^4$ in humans and 4.94×10^4 in mice) and Model M3 ($AIC_3 = 6.9 \times 10^4$ in humans and 5.99×10^4 in mice) incorporated only oscillations of either likelihood or prior precision. Model M4 ($AIC_4 = 9.8 \times 10^4$ in humans and 9.19×10^4 in mice) lacked any oscillations of likelihood and prior precision and corresponded to the normative model proposed by Glaze et al.[51]. In model M5 ($AIC_4 = 1.16 \times 10^5$ in humans and 1.14×10^5 in mice), we furthermore removed the integration of information across trials, such that perception depended only in incoming sensory information.

- B. Subject-level AIC. Here, we show the distribution of AIC values at the subject-level. AIC for the bimodal inference model tended to be smaller than AIC for the comparator models (statistical comparison to the second-best model M2 in humans: $\beta = -1.81 \pm 0.27$, $T(5.07 \times 10^3) = -6.82$, $p = 1.02 \times 10^{-11}$; mice: T(1.5^{3}) = -6.28, p = 4.38×10^{-10}).

In light of our response to Comments 1 - 9 of Reviewer 1, we agree that a complete and extensive investigation of the relation between bimodal inference, learning about changes in the environment and the relation to metacognition may be beyond the scope of the current manuscript: Both Reviewer 1 and 3 (see below) have shared that the manuscript is too long and should be streamlined. Yet evaluating the full model space (e.g., comparing update rules for inferences about H and M, testing for an influence of beliefs about M on learning about H etc.) would make the manuscript even longer. We are therefore happy to follow Prof. Fristons suggestions to omit section 5.8. We have changed the manuscript in the following ways:

When introducing ω_{LLR}, we identify it as the precision afforded to the likelihood, referring to the Bayesian framework, and refer to fluctuations in mode as a hyperprior.

- To allow for *bimodal inference*, i.e., alternating periods of internally- and externally-biased modes of perceptual processing that occur irrespective of the sequence of preceding experiences, we assumed that likelihood and prior vary in their influence on the perceptual decision according to fluctuations governed by ω_{LLR} and ω_{ψ}. These anti-phase sine functions (defined by amplitudes $a_{LLR/\psi}$, frequency f and phase p) determine the precision afforded to the likelihood and prior[53]. The implicit anti-phase fluctuations are mandated by Bayes-optimal formulations in which inference depends only on the relative values of prior and likelihood precision (i.e., the Kalman gain[54]). As such, ω_{LLR} and ω_{ψ} implement a hyperprior[55] in which the likelihood and prior precisions are shifted against each other at a dominant timescale defined by f.

We have deleted the section 5.8 and added a summary of our modeling approach to the discussion, closely following the text recommended by Prof. Friston:

- (…) Yet relying too strongly on serial dependencies may come at a cost: When accumulating over time, internal predictions may eventually override external information, leading to circular and false inferences about the state of the environment[57]. Akin to the wake-sleep-algorithm in machine learning[58], bimodal inference may help to determine whether errors result from external input or from internally-stored predictions: During internal mode, sensory processing is more strongly constrained by predictive processes that auto-encode the agent's environment. Conversely, during external mode, the network is driven predominantly by sensory inputs[18]. Between-mode fluctuations may thus generate an unambiguous error signal that aligns internal predictions with the current state of the environment in iterative test-update-cycles[58]. On a broader scale, between-mode fluctuations may thus regulate the balance between feedforward versus feedback contributions to perception and thereby play a adaptive role in metacognition and reality monitoring[59].

- We hypothesized that observers have certain hyperpriors that are apt for accommodating fluctuations in the predictability of their environment, i.e., people believe that their world is inherently volatile. To be Bayes optimal, it is therefore necessary to periodically re-evaluate posterior beliefs about the parameters that define an internal generative model of the external sensory environment. One way to do this is to periodically suspend the precision of prior beliefs and increase the precision afforded to sensory evidence, thus updating Bayesian beliefs about model parameters.

- The empirical evidence above suggests that the timescale of this periodic scheduling of evidence accumulation may be scale-invariant. This means that there may exist a timescale of periodic fluctuations in precision over every window or length of perceptual decision-making. Bimodal inference predicts perceptual decisions under a generative model (based upon a hazard function to model serial dependencies between subsequent trials) with periodic fluctuations in the precision of sensory evidence relative to prior beliefs at a particular timescale. Remarkably, a systematic model comparison based on AIC indicated that a model with fluctuating precisions has much greater evidence, relative to a model in the absence of fluctuating precisions. This ad-hoc addition of oscillations to a normative Bayesian model of evidence accumulation[51] allowed us to quantify the dominant timescale of periodic fluctuations mode at approximately 0.11 $1/N_{trials}$ in humans and mice that is appropriate for these kinds of paradigms.

Following the deletion of section 5.8, we have adapted the last sentence of the abstract and the last paragraph of the introduction:

- (…) We propose that between-mode fluctuations generate unambiguous error signals that enable optimal inference in volatile environments.

- When less sensitive to external stimulus information, humans and mice did not behave more randomly, but showed stronger serial dependencies in their perceptual choices[22–33].** These serial dependencies may be understood as driven by internal predictions that reflect the autocorrelation of natural environments[34] and bias perception toward preceding experiences[30,31,35]. Computational modeling indicated that ongoing changes in perceptual performance may be driven by systematic fluctuations between externally- and internally-oriented modes of sensory analysis. **We suggest that such *bimodal inference* may help to build stable internal representations of the sensory environment despite an ongoing stream of sensory information.

Comment 11

This begs the question as to whether you want to pursue the 1/f story. You refer to this as "noise". However, there is no noise in this setup. I think what you meant was that the fluctuations are scale free, because they evinced a power law. I am sure that there are scale free aspects of these kinds of hyperpriors; however, in the context of your paradigm I wonder whether you should just ignore the scale free aspect and focus on

your estimated temporal scale implicit in f. This means you don't have to hand wave about self-organized criticality in the discussion and focus upon your hypothesis.

We would like to thank the reviewer for this suggestion. We agree that the discussion of self-organized criticality is far from the data. We have omitted this section from the discussion. With respect to f, we have adapted the manuscript to make clear that it captures the dominant timescale of fluctuations in mode:

- (…) This implements a hyperprior[55] in which the likelihood and prior precisions are shifted against each other at a dominant timescale defined by f: (…)
- (…) The parameter f captured the dominant time scale at which likelihood and prior precision were shifted against each other and was estimated at 0.11 $1/N_{trials}$ in both humans and mice.
- Remarkably, a systematic model comparison based on AIC indicated that a model with fluctuating precisions has much greater evidence, relative to a model in the absence of fluctuating precisions. The ad-hoc addition of oscillations to a normative Bayesian model of evidence accumulation[51] allowed us to quantify the dominant timescale of periodic fluctuations mode at approximately 0.11 $1/N_{trials}$ in humans and mice that is appropriate for these kinds of paradigms.

Comment 12

A final move—to make the paper more focused and digestible—would be to put a lot of your defensive analyses (e.g. about general arousal et cetera) in supplementary material. You have to be careful not to exhaust the reader by putting up a lot of auxiliary material before the important messages in your report.

We have followed this suggestion and move the following sections to the Supplement: section 5.3 (Internal and external modes of processing facilitate response behavior and enhance confidence in human perceptual decision-making), section 5.4 (Fluctuations between internal and external mode modulate perceptual performance beyond the effect of general response biases), section 5.5 (Internal mode is characterized by lower thresholds as well as by history-dependent changes in biases and lapses). We have also moved secondary statistics to the figure legends and to the Supplement.

Minor points

Comment 13

I cannot resist suggesting that you change your title to "Bimodal Inference in Mice and Men"

We would like to thank the Reviewer for this suggestion and agree that this would indeed sound great. However, we are worried that changing the title to *mice and men* would not be as gender-neutral as *humans and mice*. We would therefore propose to stick with the current title. If we are mistaken and *mice and men* can be considered gender-neutral (we are non-native speakers), we would be happy to change the title.

Comment 14

Please replace “infra-slow fluctuations” with “slow fluctuations”. Slow has some colloquial meaning in fMRI studies but not in any scale free context.

Done.

Comment 15

Please replace “simulated data” with “simulations” in the abstract. Finally, please replace “robust learning and metacognition in volatile environments” with “enable optimal inference and learning in volatile environments.”

Done. Since we have followed the suggestion to delete section 5.8, we have rephrased the last paragraph of the abstract into:

- (…) We propose that between-mode fluctuations generate unambiguous error signals that enable optimal inference in volatile environments.

Comment 16

Line 50, please replace “about the degree of noise inherent in encoding of sensory information” with “the precision of sensory information relative to prior (Bayesian) beliefs.”

Done.

Comment 17

Line 125: please replace “a source of error” with “a source of bias”

Done.

Comment 18

Line 141: please replace “one 1/f noise” with a scale-invariant process with a 1/f power law” (here and throughout) this is not “noise” it is a particular kind of fluctuation.

Done.

Comment 19

Line 178, when you say that the fluctuations may arise due to “changes in level of tonic arousal or on-task attention”, I think you need to qualify this. In predictive processing, on-task attention is exactly the modulation of sensory precision, relative to prior precision that you are characterizing here. Tonic arousal may be another thing may or may not confound your current results.

Thank you very much for pointing this out. We have adapted the discussion to make the distinction between attention in the predictive processing sense and the broader issue of

task engagement (reflecting fluctuations in arousal, fatigue etc.) clearer (see also our responses above):

- As a functional explanation for bimodal inference, we propose that perception temporarily disengages from internal predictions to form stable inferences about the statistical properties of the sensory environment. Between-mode fluctuations may thus elude circular inferences that occur when both the causes and the encoding of sensory stimuli are volatile[19,57]). By the same token, we suggest that fluctuations in mode occur at the level of perceptual processing[26,30,46,47], and are not a passive phenomenon that is primarily driven by factors situated up- or downstream of sensory analysis.

- How does attention relate to between-mode fluctuations? According to predictive processing, attention corresponds to the precision afforded to the probability distributions that underlie perceptual inference[53]. As outlined above, between-mode fluctuations can be understood as ongoing shifts in the precision afforded to likelihood (*external mode*) and prior (*internal mode*), respectively. When the precision afforded to prior or likelihood increases, posterior precision increases, which leads to faster RTs and higher confidence. When defined from the perspective of predictive processing as the precision afforded to likelihood and prior[53], fluctuations in attention may thus provide a plausible explanation for the quadratic relationship between mode and RTs and confidence (Figure 2H and J; Figure 3I; Figure 3I).

- Outside of the predictive processing field, attention is often understood in the context of task engagement[63], which varies according to the availability of cognitive resources that are modulated by factors such as tonic arousal, familiarity with the task, or fatigue[63]. Our results suggest that internal mode processing cannot be completely reduced to intervals of low task engagement: In addition to shorter RTs and elevated confidence, choices during internal mode were not random or globally biased, but driven by perceptual history (Supplemental Figures S6-7). Moreover, our computational model identified the dominant timescale of between-mode fluctuations at 0.11 $1/N_{trials}$, which may be compatible with fluctuations in arousal[64], but is faster than to be expected for the development of task familiarity or fatigue.

- However, in interpreting the impact of between-mode fluctuations on perceptual accuracy, speed of response and confidence, it is important to consider that global modulators such as tonic arousal are known to have non-linear effects on task performance[65]: In perceptual tasks, performance seems so be highest during mid-level arousal, whereas low- and high-level arousal lead to reduced accuracy and slower responses[65]. This contrasts with the effects of bimodal inference, where accuracy increases linearly as one moves from internal to external mode, and responses become faster at both ends of the mode spectrum.

- Of note, high phasic arousal has been shown to suppress multi-domain biases in decision-making in humans and mice[66–68], including the biases toward perceptual history[28] that we implicate in internal mode processing. The increase in response

speed and history congruence over time (Supplemental Section 1.4) may argue against insufficient training as an alternative explanation for internal mode processing, but may also taken as a sign of waning arousal. The multiple mechanistic mappings to RTs and confidence therefore warrant more direct measures of arousal (such as pupil size[28,65,66,68–70], motor behavior[69,70], or neural data[71]) to better delineate bimodal inference from fluctuations in global modulators of task performance.

Comment 20

When introducing Equation 2, please make it clear that the omega terms stand in for the precisions afforded to the likelihood (omega_LLR) and prior (omega_ψ) that constitute the log posterior.

We have modified the introduction of equation 2 as follows:

- Following Bayes' theorem, we reasoned that binary perceptual decisions depend on the log posterior ratio L of the two alternative states of the environment that participants learn about via noisy sensory information[51]. We computed the posterior by combining the sensory evidence available at time-point t (i.e., the log likelihood ratio LLR) with the prior probability ψ**, weighted by the respective precision terms ω_{LLR} and ω_{ψ}:

You can then motivate Equation 6 and 7 as implementing the hyperprior in which the sensory and prior precisions fluctuate at a particular time scale.

We would like to thank the reviewer for this suggestion, which we have added to the introduction of equations (6) and (7):

- To allow for *bimodal inference*, i.e., alternating periods of internally- and externally-biased modes of perceptual processing that occur irrespective of the sequence of preceding experiences, we assumed that likelihood and prior vary in their influence on the perceptual decision according to fluctuations governed by ω_{LLR} and ω_{ψ}. These anti-phase sine functions (defined by amplitudes $a_{LLR/\psi}$, frequency f and phase p) determine the precision afforded to the likelihood and prior[53]. The implicit anti-phase fluctuations are mandated by Bayes-optimal formulations in which inference depends only on the relative values of prior and likelihood precision (i.e., the Kalman gain[54]). As such, ω_{LLR} and ω_{ψ} implement a hyperprior[55] in which the likelihood and prior precisions are shifted against each other at a dominant timescale defined by f: (…)

Comment 21

You can also point out that the implicit anti-phase fluctuations are mandated by Bayes optimal formulations in which it is only the relative values of the prior and sensory precision that matter. Bayesian filters these precisions constitute the Kalman gain. You can find a derivation of why this in treatments of the hierarchical Gaussian filter is by Mathys et al.

We would like to thank the reviewer for this suggestion. We added this information to the description of our model in the Results section (see comment above).

Comment 22

In your first model simulations, I would make it clear in the main text which parameters you are optimizing's; namely (H, alpha, a_likelihood, a_prior f). Perhaps a little table with a brief description of the meaning of these hyper parameters would be useful?

We now identify the optimized parameters at the outset of the modeling section:

- (…) We used a maximum likelihood procedure to fit the bimodal inference model (M1, Figure 1F) to the behavioral data from the Confidence database[20] and the IBL database[21], optimizing the parameters α, H, amp_{LLR}, amp_{ψ}, f, p and ζ (see Methods for details and Supplemental Table T2 for a summary of the parameters of the bimodal inference model). We validated our model in three steps: (…).

We furthermore included a table summarizing the model parameters in the Supplement (Supplemental Table T2).

Comment 23

Please remove Section 5.8. If you do not, you need to explain why — on line 586 - setting a = 0 is appropriate when a = 0, the log posterior in Equation 2 is zero because the precisions (omegas) are zero (by Equations 6 and 7).

We have removed the section 5.8. When setting the amplitude parameters to zero, ω_{LLR} and ω_{ψ} are constant at 1, creating a unimodal control model that corresponds to the normative Bayesian evidence accumulation model proposed by Glaze et al[51].

Reviewer 2

The authors elucidate whether periodicities in the sensitivity to external information represent an epiphenomenon of limited processing capacity or, alternatively, result from a structured and adaptive mechanism of perceptual inference. Analyzing large datasets of perceptual decision-making in humans and mice, they investigated whether the accuracy of visual perception is constant over time or whether it fluctuates. The authors found significant autocorrelations on the group level and on the level of individual participants, indicating that a stimulus-congruent response in a given trial increased the probability of stimulus-congruent responses in the future. Furthermore, the authors addressed whether observers cycle through periods of enhanced and reduced sensitivity to external information or whether observers rely on internal information in certain phases. This was quantified by whether a response at a given trial was correlated with responses in previous trials. The authors used computational modeling to infer the origin of the different modes (internal vs. external).

Evaluation

This is a very interesting and well-written manuscript, dealing with an important question. The findings are novel and provide an innovative account of interpreting visual perception. I am not an expert in modeling, so I will restrict my comments to theoretical framework and the experimental approach. I have a few minor questions that I would like the authors to answer or clarify.

We would like to thank the reviewer for the evaluation of our manuscript. We have added the discussion of potential effects at the motor-level to our discussion.

Minor questions

Comment 1

History congruent perception was defined on the basis of response repetitions. Are we really sure that responses are repeated due to some variant of a perceptual decision process (internal or external) or may arise on the motor-level - independent of a perceptual source? For instance, a response primed by residual activation in the motor system may represent a local effect independent from a general response bias. If indeed, a response repetition is initiated by whatever reasons (non-perceptual), wouldn't this imply that the repeated response is per se more related to previous than to current visual information and would hence signal a reduced sensitivity to current external information? The authors are discussing the option of stereotypically repeated responses in the context of alertness. However, a tendency to repeat responses may arise due to other reasons. For instance, may the motor priming effects mentioned possibly explain faster RTs along with a stronger bias when in internal-mode.

Thanks a lot for pointing this out. In this manuscript, we attempt to characterize the phenomenon of bimodal inference at the level of behavior: The Confidence database consists only of behavioral data. At the time of publishing this paper as a preprint, the IBL database had also released only behavioral data.

We realize that it is very difficult to preclude all influences from effects that occur at the level of behavior. Not all studies in the confidence database have used a counter-balanced mapping between the perceptual decision and the associated motor-response. In the IBL data, the mapping between the perceptual decision and the associated motor-response is fixed (turning a response wheel left or right depending on the perceived location of a grating). Confidence reports also provide only indirect information on motor- vs. perceptual effects. One may speculate that, if a response was driven by residual activity in the motor system, it may be more likely to be a lapse and be accompanied by reduced confidence. By contrast, we found that confidence was, on average, elevated for history-congruent choices.

That being said, behavioral analyses alone are insufficient to rule out the contribution of motor-related effects to seriality in choices. This would require analyses of additional types of data, such as video tracking of the motor response or even neural data collected in brain areas directly related to motor behavior. While this analysis is beyond the scope of the present manuscript, we plan to carry out these analyses using the recent data publication of the IBL, that contains, among others, video tracking of the motor response (turning of the

response wheel) and neuropixel recording across the whole brain, including premotor and motor cortex[71].

We have added these considerations to the discussion of potential confounds:

- Residual activation of the motor system may provide another contribution to serial biases in perceptual choices[72]. Such motor-driven priming may lead to errors in randomized psychophysical designs, resembling the phenomenon that we identify as internally-biased processing[73]. Moreover, residual activation of the motor system may lead to faster responses, and thus constitutes an alternative explanation for the quadratic relationship of mode with RTs[72]. The observation of elevated confidence for stronger biases toward internal mode speaks against the proposition that residual activation of the motor system is the primary driver of serial choice biases, since strong motor-driven priming should lead to frequent lapses that are typically associated reduced confidence[74]. Likewise, perceptual history effects have repeatedly been replicated in experiments with counter-balanced stimulus-response mappings[82].

- **No-response paradigms, in which perceptual decision are inferred from eye-movements alone, could help to better differentiate perceptual from motor-related effects. Likewise, video-tracking of response behavior and neural recording from motor- and premotor, which has recently been released for the IBL database[71], may provide further insight into the relation of motor behavior to the perceptual phenomenon of between-mode fluctuations.**

Reviewer 3

In this paper the authors propose that during perceptual decisions, humans and mice exhibit regular oscillatory fluctuations between an "external" (that places more weight on the perceptual evidence) and an "internal" (that places more weight on historical experiences) mode. In particular, the authors propose a computational scheme in which the influences of history and current stimulus on choice oscillate in anti phase, effectively implementing "bimodal inference". The computational advantages of these scheme as well as its relation to the underlying neurophysiology are discussed.

Overall, the authors make a very interesting proposal about what drives slow fluctuations in perceptual performance during randomized two-alternative choice tasks. This proposal relates changes in accuracy with changes in serial choice biases, which is a timely and synthesizing contribution. Furthermore, this proposal is backed by analyses over several human datasets and a large dataset in mice.

Despite its strong empirical contribution, the paper seems limited by the fact that alternative computational hypotheses are not adequately considered (or at least considered in a systematic way). At the same time, and although the paper is well written, some parts are overly technical.

We would like to thank the Reviewer for the very helpful comments on our manuscript. We fully agree that the previous version of our manuscript did not consider alternative computational hypotheses in a systematic and adequate way. As we outline in more detail in our point-by-point-responses below, we have addressed this issue by adding a formal model comparison of the bimodal inference model to reduced models, including a normative models of Bayesian evidence accumulation. We have added null-hypothesis-testing for enhanced history-congruence during internal mode. We have also moved a number of defensive analyses to the supplement.

Major comments:

Comment 1

The authors collapse across various datasets in which different tasks were employed. However, some details on the nature of these different tasks and a discussion on the rationale of collapsing behavioral metrics across them is missing. The authors mention that all tasks involved binary perceptual decisions. In some parts of the manuscript the term "false alarms" is mentioned, indicating a detection protocol. Other terms in the methods section (e.g., "set size") might need further clarification. Importantly, it is not clear how reaction times were calculated in the various tasks and whether some experiments involved free response paradigms while others interrogation/ cued paradigms (in which case RTs can be defined as the latency between the response cue and the response).

We would like to thank the Reviewer for this important point. Regarding the rationale for collapsing across the studies in the Confidence database: Having found strong evidence of apparent between-mode fluctuations in a study on intermittent bistable perception[19], our goal was to test whether between-mode fluctuations were a general phenomenon in perceptual decision-making. We thus had a lenient threshold for including studies from the Confidence database, i.e., all that addressed the domain of perception in 2AFC tasks. This has the advantage of looking at perceptual 2AFC decisions in general and indicated that history effects and bimodal inference are present in a wide variety of tasks.

At the same time, collapsing across diverse set of experiments means that the stimuli, the timing and the way that responses were collected differed between them. To account for this variability, we included individual experiments as random factors in linear mixed modeling. We also made sure that perceptual performance is comparable across studies (Supplemental Figure S1A-B). While our analysis of the IBL database was mainly motivated by investigating bimodal inference across species, it also allowed us to replicate our results in a highly standardized task that was collected across many individual subjects[21]. We have added the rationale for collapsing across studies with its advantages and disadvantages to the *Open questions and limitations* subsection of the discussion:

- Our results suggest bimodal inference as a pervasive aspect in perceptual decision-making in humans and mice. However, a number of limitations and open questions have to be considered: First, this work sought to understand whether fluctuations between internal and external mode, which we initially observed in an experiment on bistable perception in humans[19], represent a general phenomenon that occurs across

a diverse set of perceptual decision-making tasks. Our analysis of the Confidence database[20] therefore collapsed across all available experiments on binary perceptual decision-making. Individual experiments differed with respect to the stimuli, the manipulation of difficulty, the timing of trials, and the way responses were collected, but were highly comparable with respect to the central variables of stimulus- and history-congruence (Supplemental Figure S1A-B).

- The variability across experiments, which we considered as random effects in all statistical analyses, enabled us to assess whether bimodal inference represents a general phenomenon in perceptual decision-making, but limited the precision at which we were able to investigate the relation of mode to behavioral variables such as timing, task difficulty, RT or confidence. This issue is partially resolved by our analyses of the IBL database, which replicated our findings in an experiment that was highly standardized with respect to timing, task difficulty, and behavioral read-out[21]. It will be an important task for future research to validate our results on bimodal inference in a standardized dataset of comparable volume in humans, which is, to our knowledge, not yet available.

We apologize for the lack of clarity regarding the way we queried the Confidence database. To select a broad variety of experiments on 2AFC perceptual decision-making, we queried the Confidence database for studies from the *perception* category (excluding studies from the categories *cognitive*, *motor*, *memory* and *mixed*) and selected studies with 2AFC responses. Our previous version of the manuscript had mentioned the variable names given to the difficulty variable in the individual experiments (i.e., the name of the column in the individual *.txt* files provided for every experiment in the Confidence database). The term *false alarm* turned up in the discussion of lapses of attention and arousal, which we have re-written in response to Comment 19 by Reviewer 1 and Comment 6 by Reviewer 3. We now provide more information on the experiments and variables selected in the Method section:

- We downloaded the human data from the Confidence database[20] on 10/21/2020, limiting our analyses to the category *perception*. Within this category, we selected studies in which participants made binary perceptual decisions between two alternatives. We excluded two experiments in which the average perceptual accuracy fell below 50%. After excluding these experiments, our sample consisted of 21.05 million trials obtained from 4317 human participants and 66 individual experiments (Supplemental Table 1). Out of the 66 included experiments, 62 investigated visual, 1 auditory, 2 proprioceptive, and 1 multimodal perception. 59 experiments were based on discrimination and 6 on detection, with one investigating both.

- Out of the 58 experiments that provide information on RTs, 46 cued the response by the onset of a response screen or an additional response cue, whereas 14 allowed participants to respond at any time after stimulus onset.

- 21 of the 66 included experiments used fixed difficulty levels, whereas 45 manipulated difficulty levels within participants. Difficulty was manipulated via noise masks, contrast, luminance, presentation time, or stimulus probability for gabors, dot coherence for random dot kinematograms, difference in elements and set size for

comparisons of numerosity, difference in clicks for auditory discrimination, temporal distance for meta-contrast masking, and amount of self-motion for proprioception. We treated task difficulty as a missing variable for the experiments that fixed it at the participant-level, as this precluded the computation of autocorrelation curves.

Comment 2

The key premise that when participants do not rely on the external stimulus they rely more on the previous trial needs to be more clearly (and statistically) contrasted against a null hypothesis. For instance, an null hypothesis could be that when participants place a lower weight on the stimulus they simply choose randomly. It is important to specify a null hypothesis such that the key premise does not appear self-evident or circular.

We would like to thank the reviewer for highlighting this important point. Following this suggestion, we have explicitly tested our main hypothesis ($H1$: periods of reduced stimulus-congruence are periods of enhanced reliance on history-congruence) against the following null hypotheses:

- $H0_1$: Periods of reduced stimulus-congruence are periods of enhanced random choices
- $H0_2$: Periods of reduced stimulus-congruence are periods of enhanced general bias

We present three sets of statistical analyses to test H1 against $H0_{1/2}$:

First, we used logistic regression to predict individual choices. Under H1, one would expect a significant effect of perceptual history in a logistic regression model that predicts individual choices from the external stimulus, perceptual history and general response bias. At the model level, one would expect higher AIC in a model without perceptual history as a predictor of individual choices, indicating that perceptual history influences choices beyond noise ($H0_1$) and general response bias ($H0_2$).

In both humans and mice, we found a significant effect of perceptual history on choices while controlling for bias. When eliminating perceptual history as a predictor of individual choices, we found higher AIC (providing model-level evidence against $H0_1$ and $H0_2$). We complemented this analysis by computing AIC in individual observers (Supplemental Figure S4), and again found higher AIC in models from which perceptual history was eliminated. We have made the following changes to the main manuscript:

Humans:

- Subsection title: Fluctuations between internal and external mode cannot be reduced to general response biases or random choices

- The core assumption of bimodal inference - that ongoing changes in the sensitivity to external information are driven by internal predictions induced via perceptual history - needs to be contrasted against two alternative hypotheses: When making errors, observers may not engage with the task and respond stereotypically, i.e., exhibit stronger general biases toward one of the two potential outcomes, or simply

choose randomly. Logistic regression confirmed that perceptual history made a significant contribution to perception (β = 0.11 ± 5.79×10^{-3}, z = 18.53, p = 1.1×10^{-76}) over and above the ongoing stream of external sensory information (β = 2.2 ± 5.87×10^{-3}, z = 375.11, p = 0) and general response biases toward (β = 15.19 ± 0.08, z = 184.98, p = 0). When eliminating perceptual history as a predictor of individual choices at individual trials, AIC increased by δ_{AIC} = 1.64^{3} (see Supplemental Figure S4A-B for parameter- and model-level inference at the level of individual observers).

Mice:

- In line with humans, mice were biased toward perceptual history in 54.03% ± 0.17% of trials (T(163) = -7.52, p = 3.44×10^{-12}; Figure 4A and Supplemental Figure S1D). Perceptual history effects remained significant (β = 0.51 ± 4.49×10^{-3}, z = 112.84, p = 0) when controlling for external sensory information (β = 2.96 ± 4.58×10^{-3}, z = 646.1, p = 0) and general response biases toward one of the two potential outcomes (β = -1.78 ± 0.02, z = -80.64, p = 0). When eliminating perceptual history as a predictor of individual choices, AIC increased by δ_{AIC} = 1.48^{4}, arguing against the notion that choice randomness and general response bias are the only determinants of perceptual performance (see Supplemental Figure S4C-D for parameter- and model-level inference within individual mice).

Second, we analyzed dynamic changes in history- and stimulus-congruence (i.e., smoothed probabilities for stimulus-congruence, history-congruence and general response bias in sliding 10 trial time-windows). Under H1, one would expect a significant negative correlation between the dynamic probability of stimulus- and history-congruence. At the model level, one would expect higher AIC in a model without history-congruence as a predictor of stimulus-congruence, indicating that changes in the probability of history-congruence influence stimulus-congruence beyond noise ($H0_1$) and general response bias ($H0_2$).

In both humans and mice, we found a significant negative correlation between history-congruence and stimulus-congruence while controlling for general response bias. When eliminating the dynamic probability of history-congruence as a predictor of stimulus-congruence, we found higher AIC (providing model-level evidence against $H0_1$ and $H0_2$). The section 5.4 or our original manuscript (*Fluctuations between internal and external mode modulate perceptual performance beyond the effect of general response biases*) complements these control analyses and has been moved to the supplement to stream-line the manuscript (following the Comment 12 by Reviewer 1 and Comment 9 by Reviewer 3). We have modified the main manuscript in the following way:

Humans:

- Finally, we ensured that fluctuations in stimulus- and history-congruence are linked to each other, while controlling for fluctuations in the strength of general response biases. When perceptual choices were less biased toward external information, participants relied more strongly on internal information acquired from perceptual history (and vice versa, β = -0.05 ± 5.63×10^{-4}, T(2.1×10^{6}) = -84.21, p = 0),

controlling for fluctuations in the strength of general response biases ($\beta = -0.06 \pm 5.82 \times 10^{-4}$, $T(2.1 \times 10^{6}) = -103.51$, p = 0).

- (…) Likewise, eliminating the dynamic fluctuations in history-congruence as a predictor of fluctuations in stimulus-congruence yielded an increase in AIC by _{AIC} + 7.06^{3}. These results provided model-level evidence against the null hypotheses that fluctuations in stimulus-congruence are driven exclusively by choice randomness or general response bias (see Supplemental Section 1.2 for an in-depth assessment of general response bias).

Mice:

- As in humans, fluctuations in the strength of history-congruent biases had a significant effect on stimulus-congruence ($\beta_1 = -0.12 \pm 7.17 \times 10^{-4}$, $T(1.34 \times 10^{6}) = -168.39$, p = 0) beyond the effect of ongoing changes in general response biases ($\beta_2 = -0.03 \pm 6.94 \times 10^{-4}$, $T(1.34 \times 10^{6}) = -48.14$, p = 0). Eliminating the dynamic fluctuations in history-congruence as a predictor of fluctuations in stimulus-congruence resulted in an increase in AIC by _{AIC} + 2.8^{4}. This confirmed that, in both humans and mice, perceptual performance is modulated by systematic fluctuations between externally- and internally-oriented modes of sensory processing that exist beyond general response bias (see Supplemental Section 1.2 for an in-depth assessment of general response bias).

Third, we analyzed full and history-conditioned psychometric curves in external and internal mode as well as across modes. Under our main hypothesis that periodic reductions in sensitivity to external information are driven by increases in the impact of perceptual history, one would expect (i) a history-dependent increase in biases and lapses (effects of perceptual history), and (ii), a history-independent increase in threshold (reduced sensitivity to external information). Conversely, if what we identified as internal mode processing was in fact driven by random choices, one would expect (i), a history-independent increase in lapses (choice randomness), (ii), no change in bias (no effect of perceptual history), and (iii), reduced thresholds (reduced sensitivity to external information). In both humans and mice, we observed the pattern predicted by H1. In response to the comments by Reviewer 1 and 3, we have significantly streamlined the manuscript and moved our assessment of psychometric functions to the supplement. We now provide a summary of our results in the main manuscript to make our reasoning with respect to $H0_1$ and $H1$ more explicit:

Humans:

- To confirm that changes in the sensitivity to external information are indicative of internal mode processing, we estimated full and history-dependent psychometric curves during internal, external, and across modes. If, as we hypothesized, internal mode processing reflects an enhanced impact of perceptual history, one would expect a history-dependent increase in biases and lapses as well as a history-independent increase in threshold. Conversely, if internal mode processing were driven by random choices, one would expect a history-independent increase in biases and threshold, and no change in bias. In line with our prediction, we found that internal mode

processing was associated with a history-dependent increase in bias and lapse as well as a history-independent increase in threshold (Supplemental Section 1.3.1 and Supplemental Figure S6). This confirms that internal mode processing is indeed driven by an enhanced impact of perceptual history.

Mice:

- When fitting full and history-conditioned psychometric curves to the data from the IBL database, we observed that internal mode processing was associated with a history-dependent increase in bias and lapse as well as a history-independent increase in threshold (Supplemental Section 1.3.2 and Supplemental Figure S7). This provided further evidence for the hypothesis that internal mode processing is driven by an enhanced impact of perceptual history, as opposed to increased choice randomness.

Comment 3

From a mechanistic (sequential sampling) perspective, several previous papers have examined whether choice history biases influence the starting point or the drift rate of the evidence accumulation process. Under the former formulation, reliance on the evidence vs. reliance on the previous choice will be naturally anti-correlated (the less weight you place on the evidence the more impactful the choice history will be, assuming that the last choice is represented as a starting point bias). This seems to be mapping onto the computational model the authors describe, in which there is a weight on the prior, a weight on the likelihood and the assumption that these weights fluctuate in anti-phase. It is not obvious that this anti-phase relationship needs to be imposed ad-hoc. Or whether it would emerge naturally (using a mechanistic or Bayesian framework). More generally, the authors assert that without an external mechanism prior biases would be impossible to overcome, and this would misfit the data. However, it would be important to a) actually show that the results cannot be explained by a single mechanism in which the anti-phase relationship is emergent rather than ad-hoc, b) relate the current framework with previous mechanistic considerations of serial choice biases.

We would like to thank the reviewer for pointing this out. We agree that both normative Bayesian and mechanistic drift diffusion are bound to lead to anti-correlated effects of sensory information and perceptual history *at the level of individual trials*. This, however, does not necessarily entail slow fluctuations in the impact of sensory information and perceptual history that evolve *over many consecutive trials*. We now provide a systematic model comparison and discuss our model in relation to drift diffusion models and descriptive models that assume slow changes in the latent parameters underlying perceptual decision-making (such as Roy et al., Neuron 2021 or Ashwood et al. Nature Neuroscience et al. 2022). We also discuss the ad-hoc nature of the bimodal inference model in the subsection *Limitations and open questions*.

- We used a maximum likelihood procedure to fit the bimodal inference model (M1, Figure 1F) to the behavioral data from the Confidence database[20] and the IBL

database[21], optimizing the parameters α, H, amp_{LLR}, amp_{ψ}, f, p, and ζ (see Methods for details and Supplemental Table T2 for a summary of the parameters of the bimodal inference model). We validated our model in three steps: First, to show that bimodal inference does not emerge spontaneously in normative Bayesian models of evidence accumulation, but requires the ad-hoc addition of anti-phase oscillations in prior and likelihood precision, we compared the bimodal inference model to four control models (M2-5, Figure 1G). In these models, we successively removed the anti-phase oscillations (M2-M4) and the integration of information across trials (M5) from the bimodal inference model and performed a model comparison based on AIC.

- Model M2 (AIC_2 = 5.7 × 10^4 in humans and 4.94 × 10^4 in mice) and Model M3 (AIC_3 = 6.9 × 10^4 in humans and 5.99 × 10^4 in mice) incorporated only oscillations of either likelihood or prior precision. Model M4 (AIC_4 = 9.8 × 10^4 in humans and 9.19 × 10^4 in mice) lacked any oscillations of likelihood and prior precision and corresponded to the normative model proposed by Glaze et al.[51]. In model M5 (AIC_4 = 1.16 × 10^5 in humans and 1.14 × 10^5 in mice), we furthermore removed the integration of information across trials, such that perception depended only in incoming sensory information (Figure 1G).

- The bimodal inference model achieved the lowest AIC across the full model space (AIC_1 = 4.73 × 10^4 in humans and 4.28 × 10^4 in mice) and was clearly superior to the normative Bayesian model of evidence accumulation (δ_{AIC} = −5.08 × 10^4 in humans and −4.91 × 10^4 in mice; Supplemental Figure S9).

- In sum, computational modeling suggested that between-mode fluctuations are best explained by two interlinked processes (Figure 1E): (i), the dynamic accumulation of information across successive trials mandated by normative Bayesian models of evidence accumulation and, (ii), ongoing anti-phase oscillations in the impact of external and internal information.

- Could bimodal inference emerge spontaneously in normative models of perceptual decision-making? In predictive processing, the relative precision of prior and likelihood determines their integration into the posterior that determines the content of perception. At the level of individual trials, the perceptual impact of internal predictions generated from perceptual history (prior precision) and external sensory information (likelihood precision) are thus necessarily anti-correlated. The same holds for mechanistic models of drift diffusion, which understand choice history biases as driven by changes in the starting point[51] or the drift rate of evidence accumulation[32]. Under the former formulation, perceptual history is bound to have a stronger influence on perception when less weight is given to incoming sensory evidence, assuming that the last choice is represented as a starting point bias. The effects of choice history in normative Bayesian and mechanistic drift diffusion models can be mapped onto one another via the Bayesian formulation of drift diffusion[60], where the inverse of likelihood precision determines the amount of noise in the accumulation of new evidence, and prior precision determines the absolute shift in its starting point[60].

- While it is thus clear that the impact of perceptual history and sensory evidence are anti-correlated *at each individual trial*, we here introduce anti-phase oscillations as an ad-hoc modification to model slow fluctuations in prior and likelihood precision that evolve *over many consecutive trials* and are not mandated by normative Bayesian or mechanistic drift diffusion models. The bimodal inference model provides a reasonable explanation of the linked autocorrelations in stimulus- and history-congruence, as evidenced by formal model comparison, successful prediction of RTs and confidence as out-of-training variables, and a qualitative reproduction of our empirical data from posterior model parameter as evidence against over- or under-fitting.

- Of note, similar non-stationarities have been observed in descriptive models that assume continuous[61] or discrete[12] changes in the latent states that modulate perceptual decision-making at slow timescales. A recent computational study[62] has used a Hidden Markov model to investigate perceptual decision-making in the IBL database[21]. In analogy to our findings, the authors observed that mice switch between temporally extended *strategies* that last for more than 100 trials: During *engaged* states, perception was highly sensitive to external sensory information. During *disengaged* states, in turn, choice behavior was prone to errors due to enhanced biases toward one of the two perceptual outcomes[62]. Despite the conceptual differences to our approach (discrete states in a Hidden Markov model that correspond to switches between distinct decision-making strategies[62] vs. gradual changes in mode that emerge from sequential Bayesian inference and ongoing fluctuations in the impact of external relative to internal information), it is tempting to speculate that engaged/disengaged states and between-mode fluctuations might tap into the same underlying phenomenon.

- A third open question concerns the computational underpinnings of bimodal inference. The addition of slow anti-phase oscillations to the integration of prior and likelihood represents an ad-hoc modification of a normative Bayesian model of evidence accumulation[51]. While the bimodal inference model is supported by formal model comparison, the successful prediction of out-of-training variables and the qualitative reproduction of our empirical data in simulations from posterior model parameters, it is an important task for future research to test (i), whether between-mode fluctuations can emerge spontaneously in hierarchical models of Bayesian inference, (ii), whether modes are continuous[19] or discrete[62], and (iii), whether bimodal inference can be causally manipulated by experimental variables. We speculate that between-mode fluctuations may separate the perceptual contribution of internal predictions and external sensory data in time, creating unambiguous learning signals that benefit inference about the precision of prior and likelihood, respectively. This proposition should be tested empirically by relating the phenomenon of bimodal inference to performance in, e.g., reversal learning, probabilistic reasoning, or metacognition.

Comment 4

The authors need to unpack their definition of history biases since in previous work biases due to the response or the identity of the stimulus at the previous trial are treated differently. Here, the authors focus on response biases but it is not clear whether they could examine also stimulus-driven history biases (in paradigms where stimulus-response is remapped on each trial).

We would like to thank the reviewer for raising this important point. We defined the history-biases reported in our main manuscript by comparing the *response about the perceived* stimulus category (A vs. B) at the current and at the preceding trial (choice history). An alternative would have been to define history biases by comparing the choice at the current trial to the stimulus category *presented* at the preceding trial (stimulus history). As we show below, perceptual responses tended to be biased not only toward choice history, but also (but to a lesser degree) to stimulus history. This is expected, as perception was *stimulus-congruent* on approximately 75% of trials, causing the effects of the preceding response and the preceding stimulus to be highly correlated. We therefore compared the effects on choice history and stimulus history induced by trials at which perception was *stimulus-incongruent*, since those trials lead to opposite predictions regarding the perceptual choice at the subsequent trial.

As expected, perceptual choices were attracted toward perceptual choices at preceding stimulus-incongruent trials (i.e., a positive effect of choice history). By contrast, perceptual choices tended to be repelled away from the stimulus presented the preceding trial. This repulsion of choices away from stimuli presented at stimulus-incongruent trials confirms that the choices at stimulus-incongruent trials were the primary driver of serial effects in perception in both humans and mice.

We now refer to our analysis on stimulus history as an additional confound, which we present in detail in the Supplement 9.1 of our manuscript:

- (…) These serial biases were effects of choice history, i.e., driven by the experiences reported at the preceding trial, and could not be attributed to stimulus history, i.e., to effects of the stimuli presented at the preceding trial (Supplemental Section 1.1).

Supplement:

- The main manuscript reports the effects of perceptual history, which we defined as the impact of the choice at the preceding trial on the choice at the current trial (henceforth *choice history*). *Stimulus history*, which is defined as the impact of the stimulus presented at the preceding trial on the choice at the present trial, represents an alternative approach to this. Here, we compare the effects of choice history to the effects of stimulus history.

- We observed a significant bias toward stimulus history (humans: 49.76% ± 0.1% of trials, β = 1.26 ± 0.94, T(373.62) = 1.34, p = 0.18; mice: 51.11% ± 0.08% of trials, T(164) = 13.4, p = 3.86×10^{-28}). The bias toward stimulus history was smaller than

the bias toward choice history (humans: β = -3.53 ± 0.5, T(66.53) = -7.01, p = 1.48×10^{-9}; mice: T(164) = -17.21, p = 1.43×10^{-38}).

- The attraction of choices toward both preceding choices and stimuli is expected, as perception was *stimulus-congruent* on approximately 75% of trials, causing choices and stimuli to be highly correlated. We therefore compared the effects of choice history and stimulus history after *stimulus-incongruent* (i.e., *error*) trials, since those trials lead to opposite predictions regarding the perceptual choice at the subsequent trial.

- As expected from the findings presented in the main manuscript, perceptual choices were attracted toward perceptual choices when the inducing trial was stimulus-incongruent (i.e., a positive effect of choice history; humans: β = $0.19 \pm 1.4 \times 10^{-4}$, z = 1.36×10^{3}, p = 0: mice: β = 0.92 ± 0.01, z = 88.82, p = 0). By contrast, perceptual choices tended to be repelled away from the stimulus presented at preceding stimulus-incongruent trial (i.e., a negative effect of stimulus history; humans: β = -0.19 ± 0.01, z = -16.47, p = 5.99×10^{-61}: mice: β = -0.92 ± 0.01, z = -88.76, p = 0). This repulsion of choices away from stimuli presented at stimulus-incongruent trials confirmed that choices (which are anti-correlated to stimuli at stimulus-incongruent trials) were the primary driver of attracting serial effects in perception.

- In sum, the above results suggest that, in both humans and mice, serial dependencies were better explained by the effects of choice history as opposed to the effects of stimulus history. This aligns with a result recently published for the IBL database, where mice were shown to follow an *action-kernel* as opposed to a *stimulus-kernel* model when integrating information across trials[81].

Comment 5

Previous work, which the authors acknowledges in their Discussion (6.5), distinguishes repetitive history biases from alternating biases. For instance, in Braun, Urai & Donner (2018, JoN) participants are split into repetitive or alternating. Shouldn't the authors define the history bias in a similar fashion? The authors point out that attracting and repelling biases operate simultaneously across different timescales. However, this is not warranted given Braun et. al and other similar papers. It is not clear how this more nuanced definition of history bias would alter the conclusions.

Our empirical results show that, on average, history biases tend to be repetitive (Figure 2A, Figure 3A, and the biases in the psychometric functions in the Supplemental Figure S6-7). In fact, only 2 of the 66 experiments we included from the Confidence database[20] showed significant alternating biases (Supplemental Figure S1, please note that history-congruence was not used in the inclusion algorithm). However, this does not rule out the possibility that there are periods of alternating biases in the other experiments with net repeating effects.

Importantly, our central finding of autocorrelation in history-congruence does not distinguish between alternating and repetitive history biases. In the plot below, we show autocorrelation curves for fluctuations in history-congruence for both alternation (hazard =

0.8) and repetition (hazard = 0.2). Both were simulated for 10 blocks of a random duration between 15 and 30 trials, interleaved with 10 blocks with no history biases (hazard = 0.5). This simulation illustrates that the autocorrelation of alternating and repeating biases is identical for symmetric pairs of hazard rates. The autocorrelation of history-congruence and the associated internal mode processing is therefore not tied to repeating biases, but accommodates alternating biases as well (which seem to be, on average and in our data, less frequent). We therefore did not separate alternating from repeating biases in our analysis of mode.

We apologize for not having recognized this aspect in the previous version of the discussion. We have rewritten the paragraph on alternating and repeating biases in the discussion, referring to the plot below, which we have added to the Supplemental Materials.

- Second, our results point to an attraction of perception toward preceding choices. Previous work has shown that perceptual decision-making is concurrently affected by both attractive and repulsive serial biases that operate on distinct time-scales and serve complementary functions for sensory processing[27,75,76]: Short-term attraction may serve the decoding of noisy sensory inputs and increase the stability of perception, whereas long-term repulsion may enable efficient encoding and sensitivity to change[27]. In the data analyzed here, history biases tended to be repetitive (Figure 2A, Figure 3A, Supplemental Figure S6 and S7), and only 2 of the 66 experiments of the Confidence database[20] showed significant alternating biases (Supplemental Figure S1). However, as we show in Supplemental Figure S14, fluctuations in both alternating and repeating history biases generate overlapping autocorrelation curves. Our analysis of between-mode fluctuations is therefore not tied exclusively to repeating biases, but accommodates alternating biases as well, such that both may lead to internally-biased processing and reduced sensitivity to external sensory information. Future work could apply our approach to paradigms that boost alternating as opposed to repeating biases, as this would help to better understand how repetition and alternation are linked in terms of their computational function and neural implementation[27].

Supplemental Figure S14

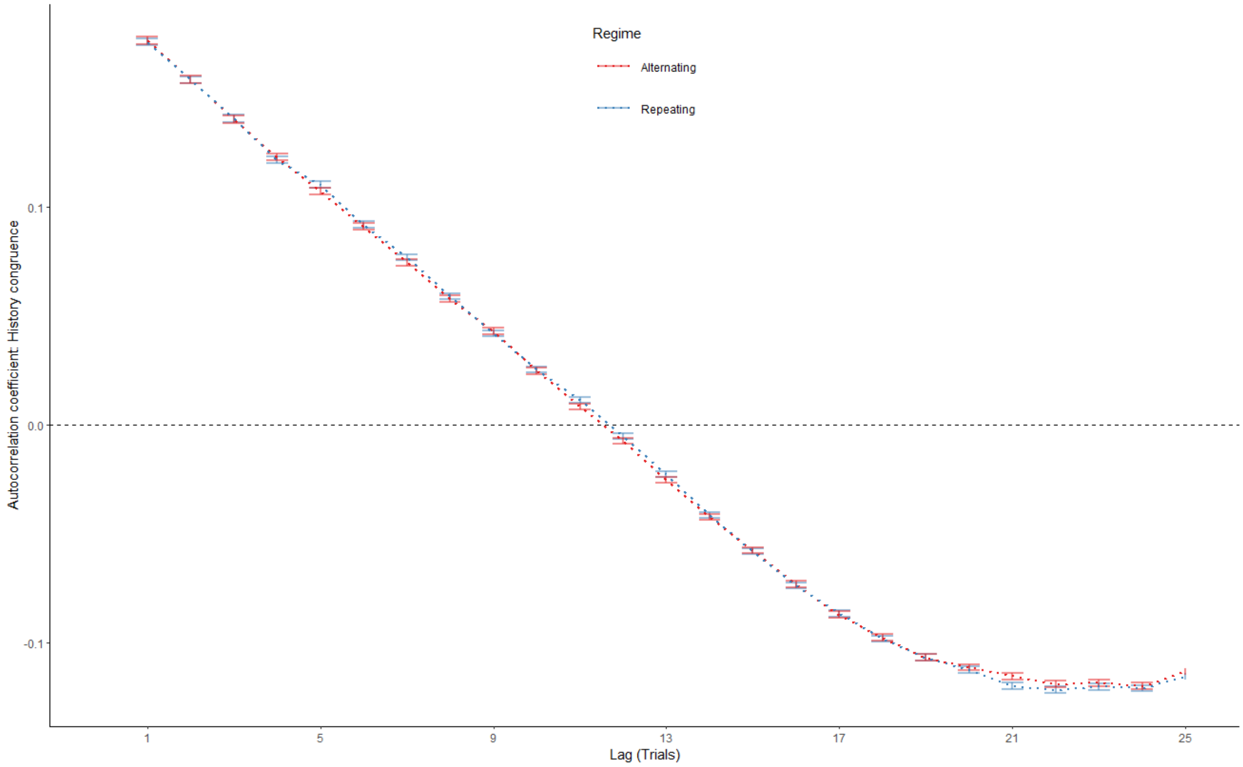

- **Supplemental Figure S14.** Autocorrelation of history-congruence of alternating and repeating biases. Here, we simulate the autocorrelation of history-congruence in 1000 synthetic participants. In the repeating regime (blue), history-congruence fluctuated between 50% and 80% (blue) in interleaved blocks (10 blocks per condition with a random duration between 15 and 30 trials). In the alternation regime (red), history-congruence fluctuated between 50% and 20%. The resulting autocorrelation curves for history-congruence overlap, indicating that our analysis is able to accommodate both repeating and alternating biases.

Comment 6

The arousal hypothesis seems to be ruled out too easily, merely in the presence of a non-monotonic "state" vs. RT pattern. Arousal can have an inverted U-shaped effect on behavioral performance and recent paper has demonstrated a non-monotonic effect of tonic arousal (baseline pupil) on RTs and accuracy (*https://www.biorxiv.org/content/10.1101/2023.07.28.550956.abstract*). More generally, the RT and confidence analyses need to be complemented, perhaps by computational modeling using sequential sampling models, as these behavioral metrics have multiple mechanistic mappings (e.g., a fast RT might correspond to high SNR or an impulsive decisions driven by a starting point bias).

We would like to thank the reviewer for this important point. Considering this Comment and the Comment 3 by Reviewer 1, we realize that the quadratic relationships between mode and RTs/confidence do not represent a convincing defensive analysis against the potential

contributions of arousal to the phenomenon that we have identified as between-mode fluctuations. Rather, we now interpret the fluctuations of RTs/confidence with mode as indicative of a scenario in which between-mode fluctuations modulate a decision-variable that determines not only the perceptual choices, but also the speed and confidence at which they are made.

Therefore, as a first response to this comment, we have re-phrased our assessment of RT and confidence in the following way:

Humans:

- The above results point to systematic fluctuations in the *decision variable*[44] that determines perceptual choices, causing enhanced sensitivity to external stimulus information during external mode and increased biases toward preceding choices during internal mode. As such, fluctuations in mode should influence downstream aspects of behavior and cognition that operate on the perceptual decision variable[44]. To test this hypothesis with respect to motor behavior and metacognition, we asked how bimodal inference relates to response times (RTs) and confidence reports. (…).
- (…) In sum, the above results indicate that reporting behavior and metacognition do not map linearly onto the mode of sensory processing. Rather, they suggest that slow fluctuations in the respective impact of external and internal information are most likely to affect perception at an early level of sensory analysis[46,47]. Such low-level processing may thus integrate perceptual history with external inputs into a decision variable[44] that influences not only perceptual choices, but also the speed and confidence at which they are made.
- In what follows, we probe alternative explanations for between-mode fluctuations, test for the existence of modes in mice, and propose a predictive processing model that explains fluctuations in mode ongoing shifts in the precision afforded to external sensory information relative to internal predictions driven by perceptual history.

Mice:

- The above results confirm that fluctuations between internally- and externally-biased modes generalize to perceptual decision-making in mice. As in humans, we hypothesized that bimodal inference modulates the decision variable[44] that determines not only perceptual choices, but also downstream aspects of mouse behavior[44]. (…). When fitting full and history-conditioned psychometric curves to the data from the IBL database, we observed that internal mode processing was associated with a history-dependent increase in bias and lapse as well as a history-independent increase in threshold (Supplemental Section 1.3.2 and Supplemental Figure S7). Over time, the frequency of history-congruent choices increased alongside stimulus-congruence and speed of response as mice were exposed to the experiment, arguing against the proposition that biases toward perceptual history reflected an unspecific response strategy in mice who were not sufficiently trained on the IBL task.

Second, we have re-structured the section of defensive analyses, where we contrast the phenomenon that we identify as fluctuating modes in perception with stereotypical or random responses as evidence of low task engagement, and not specifically with attention or arousal, which we discuss below. To streamline the manuscript, we report those analyses in detail in the Supplement (see Comments 12 by Reviewer 1 and Comment 9 by Reviewer 3).

- The core assumption of bimodal inference - that ongoing changes in the sensitivity to external information are driven by internal predictions induced via perceptual history - needs to be contrasted against two alternative hypotheses: When making errors, observers may not engage with the task and respond stereotypically, i.e., exhibit stronger general biases toward one of the two potential outcomes, or simply choose randomly. Logistic regression confirmed that perceptual history made a significant contribution to perception (β = 0.11 ± 5.79×10^{-3}, z = 18.53, p = 1.1×10^{-76}) over and above the ongoing stream of external sensory information (β = 2.2 ± 5.87 × 10^{-3}, z = 375.11, p = 0) and general response biases toward (β = 15.19 ± 0.08, z = 184.98, p = 0).

- When eliminating perceptual history as a predictor of individual choices at individual trials, AIC increased by δ_{AIC} = 1.64^{3} (see Supplemental Figure S4A-B for parameter- and model-level inference at the level of individual observers). Likewise, when eliminating slow fluctuations in history-congruence as a predictor of slow fluctuations in stimulus-congruence across trials, we observed an increase in AIC by δ_{AIC} + 7.06^{3}. These results provided model-level evidence against the null hypotheses that fluctuations in stimulus-congruence are driven exclusively by choice randomness or general response bias (see Supplemental Section 1.2 for an in-depth assessment of general response bias).

- To confirm that changes in the sensitivity to external information are indicative of internal mode processing, we estimated full and history-dependent psychometric curves during internal, external, and across modes. If, as we hypothesized, internal mode processing reflects an enhanced impact of perceptual history, one would expect a history-dependent increase in biases and lapses as well as a history-independent increase in threshold. Conversely, if internal mode processing were driven by random choices, one would expect a history-independent increase in lapses and threshold, and no change in bias. In line with our prediction, we found that internal mode processing was associated with a history-dependent increase in bias and lapse as well as a history-independent increase in threshold (Supplemental Section 1.3 and Supplemental Figure S6-7). This confirmed that internal mode processing is indeed driven by an enhanced impact of perceptual history.

- In line with this, the quadratic relationship between mode and confidence (Figure 2J) suggested that biases toward internal information do not reflect a post-perceptual strategy or repeating preceding choices when the subjective confidence in the perceptual decision is low. Moreover, while responses became faster with longer exposure to the experiments of the Confidence database, the frequency of history-congruent choices increased over time, speaking against the proposition that

participants may stereotypically repeat preceding choices when not yet familiar with the experimental task (see Supplemental Section).

- Taken together, our results thus argue against recurring intervals of low task engagement, which may be signaled by stereotypical or random responses, as an alternative explanation for the phenomenon that we identify as bimodal inference.

Third, in response the Comment 3 by Reviewer 1, we now interpret the quadratic relationship of mode to RTs/confidence in the context of predictive processing views on attention[53]. Based on the Bayesian formulation of drift diffusion[60], we propose that the effects of likelihood on prior precision on the decision variable and, consequently, on confidence and RTs can be translated into the mechanistic framework of drift diffusion. Specifically, Bitzer et al. relate likelihood precision to noise in the accumulation process, and prior precision to the amount of shift in the starting point[60]. As a third response to this comment, we have re-written our discussion of the quadratic relationship of mode to RTs and Confidence, focusing on predictive coding models attention, which we relate to mechanistic drift diffusion models:

- How does attention relate to between-mode fluctuations? According to predictive processing, attention corresponds to the precision afforded to the probability distributions that underlie perceptual inference[53]. As outlined above, between-mode fluctuations can be understood as ongoing shifts in the precision afforded to likelihood (*external mode*) and prior (*internal mode*), respectively. When the precision afforded to prior or likelihood increases, posterior precision increases, which leads to faster RTs and higher confidence. When defined from the perspective of predictive processing as the precision afforded to likelihood and prior[53], fluctuations in attention may thus provide a plausible explanation for the quadratic relationship of mode to RTs and confidence (Figure 2H and J; Figure 3I; Figure 4I). Such effects of attention in the predictive processing sense can be directly related to mechanistic drift diffusion models[60], where both larger shifts in starting point (related to increased prior precision in internal mode) and lower noise in the accumulation of evidence (related to increased likelihood precision in external mode) may explain faster and more confident responses.

Given the correspondence between normative Bayesian and mechanistic drift diffusion models[60], we believe that we would not gain additional insights into the role of arousal and additional potential confounds or causes of between-mode fluctuations by fitting drift diffusion models as an alternative class of behavioral models to our data. To understand the relation of arousal to the bimodal inference, we think that it is necessary to look at data beyond behavior, such as pupillometry, video tracking of response behavior, or neural data. While this is beyond the scope of the current manuscript, we plan to do these analyses in a follow-up paper, using data published by the IBL after this paper was submitted. The IBL data now contains eye tracking, video tracking of response behavior, and neuropixel recordings across the whole mouse brain[71]. As a fourth response to this comment, we have re-written our discussion of attention and arousal:

- Outside of the predictive processing field, attention is often understood in the context of task engagement[63], which varies according to the availability of cognitive resources that are modulated by factors such as tonic arousal, familiarity with the task, or fatigue[63]. Our results suggest that internal mode processing cannot be completely reduced to intervals of low task engagement: In addition to shorter RTs and elevated confidence, choices during internal mode were not random or globally biased, but driven by perceptual history (Supplemental Figures S6-7). Moreover, our computational model identified the dominant timescale of between-mode fluctuations at 0.11 $1/N_{trials}$, which may be compatible with fluctuations in arousal[64], but is faster than to be expected for the development of task familiarity or fatigue.

- However, in interpreting the impact of between-mode fluctuations on perceptual accuracy, speed of response and confidence, it is important to consider that global modulators such as tonic arousal are known to have non-linear effects on task performance[65]: In perceptual tasks, performance seems so be highest during mid-level arousal, whereas low- and high-level arousal lead to reduced accuracy and slower responses[65]. This contrasts with the effects of bimodal inference, where accuracy increases linearly as one moves from internal to external mode, and responses become faster at both ends of the mode spectrum.

- Of note, high phasic arousal has been shown to suppress multi-domain biases in decision-making in humans and mice[66–68], including the biases toward perceptual history[28] that we implicate in internal mode processing. The increase in response speed and history congruence over time (Supplemental Section 1.4) may argue against insufficient training as an alternative explanation for internal mode processing, but may also taken as a sign of waning arousal. The multiple mechanistic mappings to RTs and confidence therefore warrant more direct measures of arousal (such as pupil size[28,65,66,68–70], motor behavior[69,70], or neural data[71]) to better delineate bimodal inference from fluctuations in global modulators of task performance.

Comment 7

In several analysis the authors present an effect and then show that this effects persists when key variables/ design aspects are also taken into account (see an example at around line 70). It makes more sense to present only one single analysis in which these key variables are controlled for. Results cannot be interpreted if they are spurious factors driving them so it is not clear why some of the results are presented in two versions ("uncontrolled" and "controlled" analyses).

We apologize for this. We have updated our manuscript accordingly and have omitted, whenever possible, reports of uncontrolled analyses (highlighted in the main text). The most significant changes are summarized here:

- Group-level autocorrelation curves in humans: In line with previous work[8], we found that the probability of stimulus-congruence was not independent across successive trials: At the group level, stimulus-congruent perceptual choices were significantly autocorrelated for up to 15 trials (Figure 2B), controlling for task difficulty and the

sequence of presented stimuli (Supplemental Figure 2A-B). (...) In close analogy to stimulus-congruence, we found history-congruence to be significantly autocorrelated for up to 21 trials (Figure 2B), while controlling for task difficulty and the sequence of presented stimuli (Supplemental Figure 2A-B).

- Group-level autocorrelation curves in mice: At the group level, we found significant autocorrelations in both stimulus-congruence (42 consecutive trials) and history-congruence (8 consecutive trials (Figure 3B), while controlling for the respective autocorrelation of task difficulty and external stimulation (Supplemental Figure 2C-D).

- Correlation between stimulus- and history-congruence in humans: When perceptual choices were less biased toward external information, participants relied more strongly on internal information acquired from perceptual history (and vice versa, $\beta = -0.05 \pm 5.63 \times 10^{-4}$, $T(2.1 \times 10^{6}) = -84.21$, $p = 0$, **controlling for fluctuations in general response biases**; Supplemental Section 1.2).

- Correlation between stimulus- and history-congruence in mice: Fluctuations in the strength of history-congruent biases had a significant effect on stimulus-congruence ($\beta_1 = -0.12 \pm 7.17 \times 10^{-4}$, $T(1.34 \times 10^{6}) = -168.39$, $p = 0$) beyond the effect of ongoing changes in general response biases ($\beta_2 = -0.03 \pm 6.94 \times 10^{-4}$, $T(1.34 \times 10^{6}) = -48.14$, $p = 0$).

Comment 8

The central empirical finding is potentially important but is currently shadowed by more speculative sections/ discussions. For instance, the section on the adaptive merits of the computational model is relatively weaker compared to the empirical results. In particular, the model is simulated without feedback (whereas most experiments employ trial by trial feedback) and does not outperform the baseline model in accuracy but in other secondary metrics.

We agree with the Reviewer (see also a similar suggestion by Reviewer 1 in Comment 9). We have removed the section 5.8 and the associated subsection of the discussion from our manuscript. We will develop a model on the potential function of between-mode fluctuations in a separate publication.

Minor comments:

Comment 9

The amount of statistical analysis and results is often overwhelming. The authors could streamline the presentation better such that the main result is brought to the foreground. Currently the manuscript resembles a technical report.

We apologize for this. From the Results, we have moved a number of sections to the Supplemental Materials to stream-line the manuscript (i.e., our analysis of general response biases [former section 5.4], the analysis of psychometric functions [former section 5.5], and the respective paragraphs on the IBL database). From the Discussion, we have removed the

section on self-organized criticality (also following the suggestion of Reviewer 1). We have also moved more peripheral statistical results to the figure legends and the method section:

- We have moved the statistics on exponential decay in the autocorrelation to the figure legends of Figure 2B and 3B.
- We have shortened the description of logistic regression models that predict the stimulus- and history-congruence at the index trial from the stimulus- and history-congruence at the preceding trials.
- The section on mouse behavior now matches the logic and presentation of results in the human section.

We hope that these changes will increase the readability of our manuscript.

Comment 10

Some typos or omissions may alter the meaning in various places. Indicatively, in lines 273, 439, 649.

Thanks a lot, we have corrected these typos.
